# Spatial patterns of wet season precipitation vertical gradients on the Tibetan Plateau and the surroundings

**DOI:** 10.1038/s41598-017-05345-6

**Published:** 2017-07-11

**Authors:** Lan Cuo, Yongxin Zhang

**Affiliations:** 1 Center for Excellence in Tibetan Plateau Earth Sciences, Beijing, China; 2 Key Laboratory of Tibetan Environment Changes and Land Surface Processes, Institute of Tibetan Plateau Research, Chinese Academy of Sciences, Beijing, China; 3 University of Chinese Academy of Sciences, Beijing, China; 4 Research Applications Laboratory and Climate and Global Dynamics Laboratory, National Center for Atmospheric Research, Boulder, Colorado USA

## Abstract

The Tibetan Plateau and the surrounding (TPS) with its vast land mass and high elevation affects regional climate and weather. The TPS is also the headwater of 9 major Asian rivers that provide fresh water for 1.65 billion people and many ecosystems, with wet season (May–September) precipitation being the critical component of the fresh water. Using station observations, ERA-Interim and MERRA2 reanalysis, we find that wet season precipitation displays vertical gradients (i.e., changes with elevation) that vary within the region on the TPS. The decrease of precipitation with elevation occurs in the interior TPS with elevation larger than 4000 m, little or no change over the southeastern TPS, and increase elsewhere. The increase of precipitation with elevation is caused by increasing convective available potential energy (CAPE) and decreasing lifting condensation level (LCL) with elevation overwhelming the effects of decreasing total column water vapor (TCWV) with elevation. The decreasing precipitation with elevation is due to the combined effects of increasing LCL and decreasing TCWV. LCL and CAPE play a more important role than TCWV in determining the spatial patterns. These findings are important for hydrology study in observation scarce mountainous areas, water resources and ecosystem managements in the region.

## Introduction

**Figure 1:**
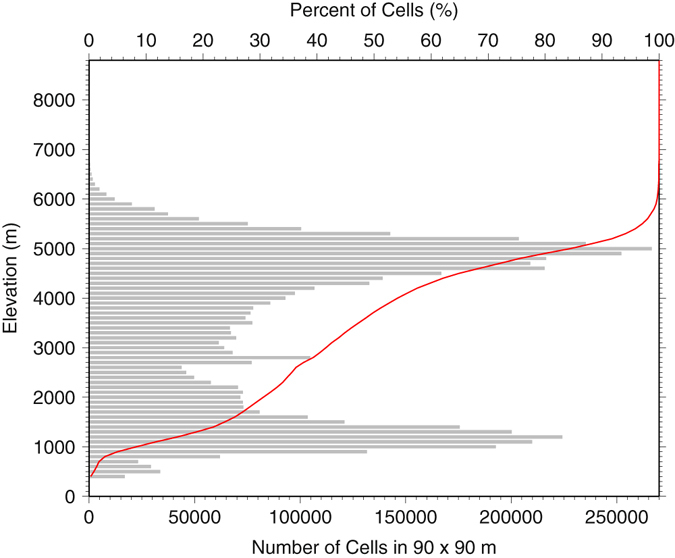
Histogram of elevation in 100 m interval (gray bars) and hypsometric curve (red line) for the Tibetan Plateau and the surroundings (TPS, 25 °N–42 °N and 77 °E–105 °E) based on the 90 m resolution digital elevation map. This figure was plotted using the Generic Mapping Tools (GMT) V4.5.0 (https://www.soest.hawaii.edu/gmt/).

The TPS, located in 25°N–42°N and 77°E–105°E, is the largest and highest plateau on Earth. Its mean elevation is approximately 4000 m based on the NASA SRTM 90 m elevation data base^1^, and 80% of the TPS is situated below 5000 m (Fig. 1). A bi-mode elevation distribution with peaks located around 1300 m and 5000 m, respectively, is evident in the histogram (Fig. 1), indicating relatively subdued topography and vast area near the two peaks. The TPS affects the subtropical westerlies, the South Asia monsoon and the East Asia monsoon through thermal and mechanical forcing, thereby exerting significant influence on regional and global climate^2–5^.

**Figure 2:**
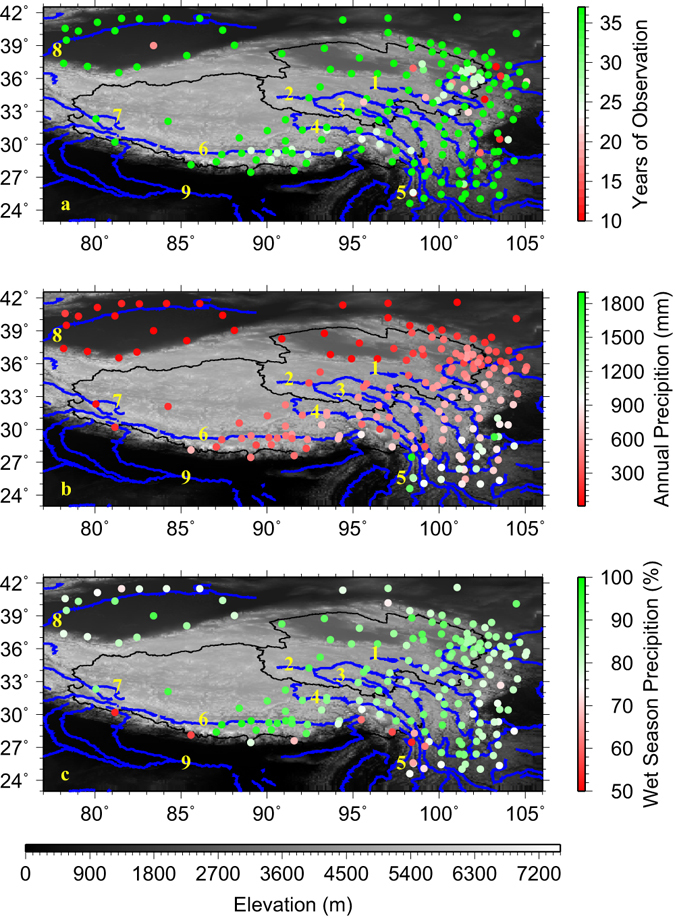
Geographic locations and number of years of observation for 185 stations that have observation periods longer than 10 years during 1979–2015 (**a**); mean annual precipitation at the 185 stations during 1979–2015 (**b**); percentage of wet season (May–September) precipitation to annual total precipitation at the 185 stations (**c**). Black lines denote the boundaries of the Qinghai Province on the northeastern and the Tibetan Autonomous Region on the southwestern TPS. Blue lines and the numerical numbers represent the 9 major rivers that originate from the TPS: 1: the Yellow River, 2: the Yangtze River, 3: the Mekong River, 4: the Salween River, 5: the Irrawaddy River, 6: the Brahmaputra River, 7: the Indus River, 8: the Tarim River, and 9: the Ganges River. This figure was plotted using the Generic Mapping Tools (GMT) V4.5.0 (https://www.soest.hawaii.edu/gmt/).

Furthermore, the TPS, often referred to as the “Water Tower of Asia”, contains the headwaters of 9 major rivers in Asia that provide vital fresh water resources for 1.65 billion people^6^ or about 22% of the world’s population as well as numerous ecosystems. The headwaters of these 9 major rivers, the Yellow, Yangtze, Mekong, Salween, Irrawaddy, Brahmaputra, Indus, Tarim and Ganges Rivers (see Fig. 2 for their locations) are mostly fed and sustained by precipitation and frozen water^7^. As frozen water contribution to fresh water resources is generally small throughout the region, precipitation remains the most important contributor to streamflow, especially in the eastern and southern river basins^7, 8^. Precipitation is also one of the most important factors that influence the succession of plant functional types on the northern TPS over the past 5 decades^9^. The observed mean annual precipitation during 1979–2015 at the stations of the TPS (Fig. 2a) ranges widely from ~20 mm in the northwest to ~1900 mm in the southeast (Fig. 2b). This southeast-northwest precipitation horizontal gradient is related to the combined effects of the East and South Asia monsoons, of the westerlies, and of the interactions between the local topography and the large-scale weather systems^10–16^. Wet season (May - September) precipitation is especially important for this region as it accounts for more than 70% of the annual total precipitation at 95% of the stations (Fig. 2c).

The generation of precipitation requires three essential elements: moisture, lifting and condensation. Lifting can be provided either by weather systems such as low-pressure systems or by topography or both, and is related to the instability of the atmosphere. Over the TPS, wet season moisture displays large spatial variations related to the circulation patterns and topography^12, 17–20^. For example, the southerly and southwesterly flows associated with the South and East Asia monsoons bring moisture laden air to the south and east TPS^12, 19^ where precipitation is relatively abundant (Fig. 2b). On the other hand, the westerlies with limited moisture influence the northwest and west TPS, the east Karakoram range, the west Himalayas and the northern slope of the plateau^4, 19^ where wet season precipitation is less than 300 mm (Fig. 2b). Clearly, the large-scale weather systems determine the regional patterns of precipitation over the TPS but the complex topography ought to exert significant influence through localizing and distributing precipitation, thus affecting the local spatial patterns of precipitation. How precipitation changes with elevation in this region and what are the controlling factors behind the changes are intriguing and at the same time critical for understanding watershed hydrology as high elevations are usually the headwaters of major rivers and have scarce observations. Due to the limited availability and scarcity of observations on the TPS in the past^21^, changes of wet season precipitation with elevation have been seldom studied and the underlying mechanisms are largely unknown. This study aims to fill the gap by making use of the recently available precipitation observations in the region and the global reanalyses as described in the Data and Method section.

## Results

**Figure 3:**
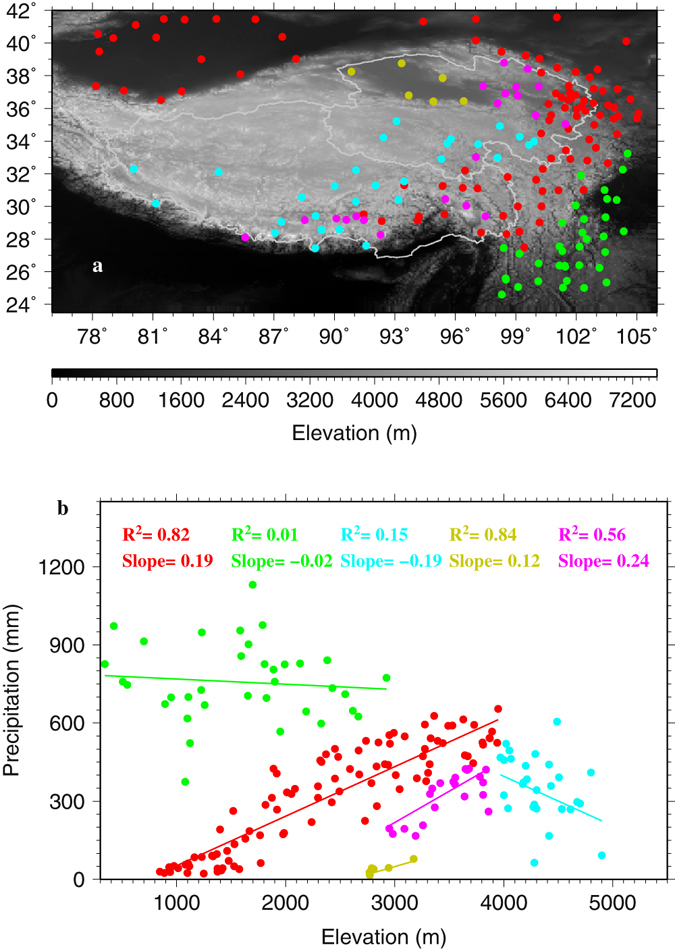
(**a**) Locations of 5 groups of stations that display different precipitation gradients with elevation identified in (**b**) on the TPS. Group Red consists of 94 stations; Group Yellow 6 stations; Group Purple 21 stations; Group Green 35 stations; and Group Blue 28 stations. This figure was plotted using the Generic Mapping Tools (GMT) V4.5.0 (https://www.soest.hawaii.edu/gmt/).

Observed mean annual wet season precipitation changes with elevation (i.e., vertical gradients) do not exhibit a clear plateau wide pattern (i.e., a slope of −0.03 mm m^−1^ and R^2^ of 0.02 for stations with elevation greater than 2000 m; Supplementary S1) but display localized distinctive clusterings over the TPS (Fig. 3b) with strong dependence on both elevation and terrain (Fig. 3a). In all, 5 groups are identified and color coded (Fig. 3 and Table 1): a) Group Red, consisting of 94 stations that are located mainly along the curved corridor of southeast-northeast-northwest section of the eastern TPS and in the upper Tarim Basin with an elevation range of 800–3900 m, shows a statistically significant increase rate of 0.19 mm m^−1^; b) Group Yellow, comprised of 6 stations located in the desert Qaidam Basin at 2700–3200 m, displays a statistically significant increase rate of 0.12 mm m^−1^; c) Group Purple, with 21 stations located in the eastern parts of the Qinghai Province and TAR (Tibet Autonomous Region) at 3000–4000 m, exhibits the highest and statistically significant increase rate of 0.24 mm m^−1^; d) Group Green, with 35 stations located in the southeast TPS at 350–3000 m, shows almost no change with elevation as reflected by a very low negative slope of −0.02 mm m^−1^ and a coefficient of determination R^2^ of 0.01; and e) Group Blue, with 28 stations located above 4000 m in the interior TPS, displays a statistically significant decrease rate of −0.19 mm m^−1^ (Table 1, Fig. 3). Both Groups Red and Yellow present a R^2^ in excess of 0.82 (Table 1). Two tailed t tests show that the slopes of these 5 groups are statistically significantly different from each other at the confidence level of 90%. For individual wet seasons during 1979–2015 (not shown), the precipitation vertical gradient patterns are very similar to those of the mean wet season gradient in 1979–2015, indicating the robustness of the identified patterns. Also, similar patters for the five groups are noticed once the stations are selected using minimum elevation thresholds of 1000, 1500, 2000 and 2500 m, respectively (see Supplementary Figures S2–S5), indicating the robustness of the classification as well.

**Table 1:** Slopes and coefficients of determination (R^2^) of the observed (Obs), ERA-Int (ERA) and MERRA2 wet season precipitation, TCWV, CAPE and LCL with respect to elevation.

Variables	Groups	Sample sizes			Slopes			R^2^		
		Obs	ERA	MERRA2	Obs	ERA	MERRA2	Obs	ERA	MERRA2
Precipitation (mm/m)^a^	Red	94	59	65	0.19*	0.32*	0.33*	0.82	0.66	0.45
	Yellow	6	6	6	0.12*	0.06*	0.11*	0.84	0.94	0.86
	Purple	21	21	18	0.24*	0.38*	0.30*	0.56	0.55	0.13
	Green	35	24	30	−0.02	0.07	0.19*	0.01	0.03	0.10
	Blue	28	22	23	−0.19*	0.20	−0.10	0.15	0.06	0
TCWV (kg m^−2^/m)^a^	Red					−0.20*	−0.22*		0.39	0.39
	Yellow					−0.36*	−0.47*		0.77	0.90
	Purple					0.13*	0.15		0.19	0.15
	Green					−1.37*	−1.41*		0.96	0.96
	Blue					−0.09	−0.26*		0.02	0.13
CAPE (J kg^−1^/m)^a^	Red					0.11*	0.01*		0.87	0.62
	Yellow					0.03	0		0.53	0.02
	Purple					0.20*	0.02*		0.75	0.52
	Green					0.02	−0.03*		0.05	0.27
	Blue					0.29*	0		0.33	0
LCL (m/m)^a^	Red					−0.59*	−0.68*		0.83	0.79
	Yellow					−0.54	−0.57*		0.48	0.94
	Purple					−0.43*	−0.41*		0.50	0.40
	Green					−0.05*	−0.05		0.14	0.07
	Blue					−0.09	0.14		0.01	0.01

The units of the slopes are denoted by^a^; *Represents statistically significant values at 90% confidence level based on the two tailed student t-test.

**Figure 4:**
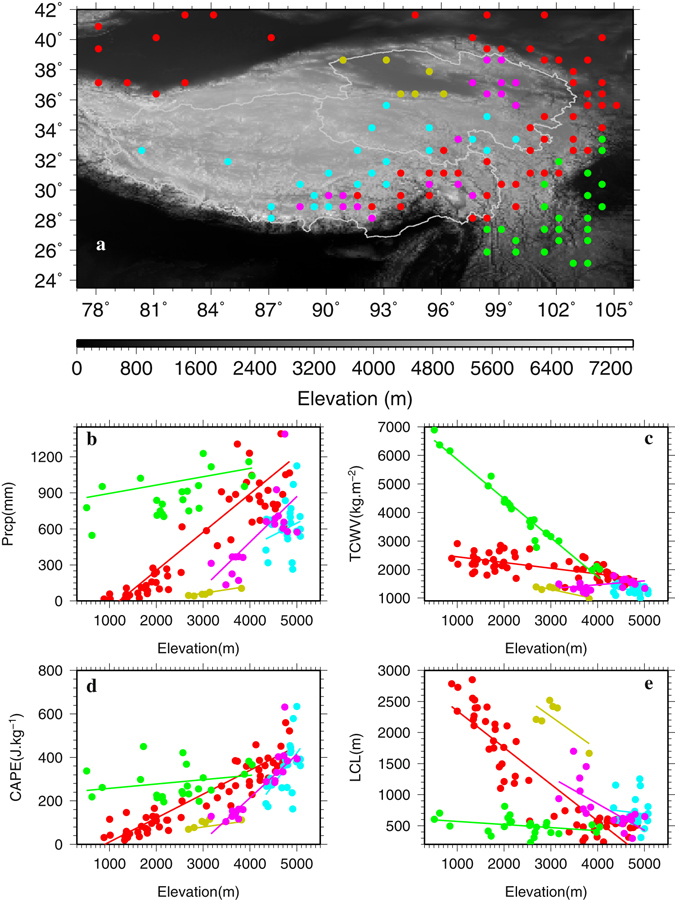
Locations of the ERA-Interim cells whose wet season precipitation is correlated statistically significantly at 90% confidence level with that of the corresponding stations (**a**). Vertical distributions of wet season precipitation (**b**), total column water vapor (TCWV) (**c**), convective available potential energy (CAPE) (**d**), and lifting condensation level (LCL) (**e**) with ERA-Interim elevation for the ERA-Interim cells that are separated into the same color-coded 5 groups as for the observations. This figure was plotted using the Generic Mapping Tools (GMT) V4.5.0 (https://www.soest.hawaii.edu/gmt/).

**Figure 5:**
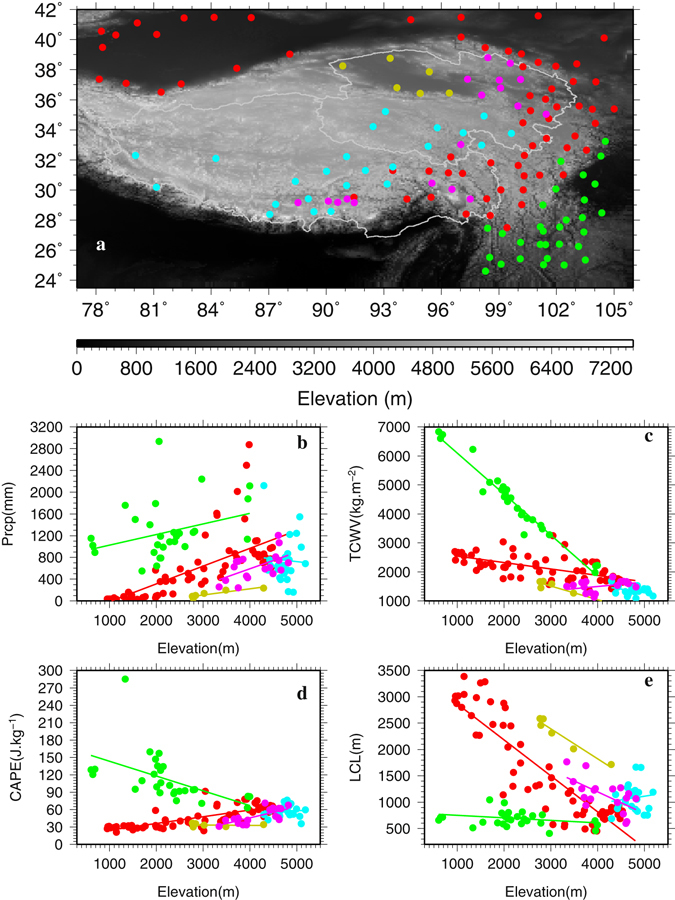
Locations of the MERRA2 cells whose wet season precipitation is correlated statistically significantly at 90% confidence level with that of the corresponding stations (**a**). Vertical distributions of wet season precipitation (**b**), total column water vapor (TCWV) (**c**), convective available potential energy (CAPE) (**d**), and lifting condensation level (LCL) (**e**) with MERRA2 elevation for the MERRA2 cells that are separated into the same color-coded 5 groups as for the observations. This figure was plotted using the Generic Mapping Tools (GMT) V4.5.0 (https://www.soest.hawaii.edu/gmt/).

To understand what factors might be responsible for the unique spatial patterns of precipitation vertical gradients over the TPS, the ERA Interim^22^ (ERA-Int) and the second-generation MERRA^23^ (MERRA2) reanalyses are utilized. Previous studies have demonstrated that ERA-Int performs as good as or even better than several other widely available reanalysis products in estimating climatology and variations of surface variables and hydrological cycle over the TPS^20, 24–26^. MERRA (and by extension MERRA2) has also been shown to perform satisfactorily over the TPS in resolving the surface meteorological variables^24^. The selection of the cells from ERA-Int and MERRA2 that match the observation sites is described in the Data and Method section. In total, 132 and 142 cells are selected for ERA-Int and MERRA2, respectively (Table 1, Figs 4a and 5a). The selected ERA-Int and MERRA2 cells exhibit generally consistent temporal variability in the normalized wet season precipitation with the observed (Supplementary Figures S8, S9).

The ERA-Int and MERRA2 cells were also divided into the same 5 groups as for the observations based on the cells’ locations that are consistent with the station locations (Figs 4a, 5a and 3a). Similarities in the scatter plots between ERA-Int (Fig. 4b) and the observations (Fig. 3b) are striking although the ERA-Int cells display a wider range of elevation and precipitation than those of the stations due to coarse resolution (~0.7°). Groups Red, Yellow and Purple for ERA-Int show increase rates of gradients as well as large R^2^ as in the observations, although for ERA-Int the increase rate of 0.32 mm m^−1^ for Group Red and 0.38 mm m^−1^ for Group Purple are much larger than the observed while the increase rate of 0.06 mm m^−1^ for Group Yellow is much smaller than the observed (Table 1). Group Green for ERA-Int displays a negligible increase rate of 0.07 mm m^−1^ and R^2^ of 0.03, indicating little change of wet season precipitation with elevation for this group, similar to the observations. A notable difference lies in Group Blue for which the observations present a decrease rate of 0.19 mm m^−1^ while ERA-Int shows an increase rate of 0.20 mm m^−1^, although the spread is large for both cases as reflected by the second smallest R^2^ in each case (Table 1). This discrepancy could be related to sparse stations in desert plains and high elevations above 4000 m as well as to insufficient horizontal resolution and possible model deficiency of ERA-Int.

For MERRA2, the wet season precipitation vertical gradients are positive for Groups Red, Yellow, and Purple, and negative for Group Blue, and these are consistent with the observations (Figs 5a and 3a, Table 1) although R^2^ is 0 for MERRA2’s Group Blue. MERRA2 has a slightly finer resolution of 0.625° longitude × 0.5° latitude than ERA-Int, which may have helped with MERRA2’s improved performance for Group Blue. However, for Group Green, MERRA2 presents a statistically significant increase rate of 0.19 mm m^−1^, in contrast to the observations and ERA-Int that show negligible vertical gradients.

The observations, ERA-Int and MERRA2 reveal a generally consistent pattern that can be summarized as follows: 1) below 4000 m and along the southwest-northeast section over the eastern TPS and in the Tarim and Qaidam Basins (Groups Red, Purple and Yellow), precipitation increases with elevation and with varying rates; 2) in the southeastern TPS where precipitation is abundant (Group Green), the observations and ERA-Int indicate little change of precipitation with elevation but MERRA2 shows a relatively large increase rate; and 3) above 4000 m, mostly in the interior of the TPS (Group Blue), the observations and MERRA2 present a decrease rate but ERA-Int displays a relatively large increase rate although for this group the spread is large as reflected by small R^2^, indicating the necessity of more observations above 4000 m.

Next we examine the wet season total column water vapor (TCWV), convective available potential energy (CAPE), and lifting condensation level (LCL) at the ERA-Int and MERRA2 cells. These three variables represent the vertically integrated moisture, instability and condensation needed for the generation and development of precipitation, with larger TCWV, higher CAPE and lower LCL favoring precipitation more. While this work will focus on the connection of the three variables with the precipitation change a future work will explore how these variables relate to the atmospheric circulations and climate systems on the TPS.

For ERA-Int, Groups Red, Yellow and Green all show statistically significant decrease rates of TCWV with elevation until around 4000 m above which TCWV becomes small and changes little with height (Fig. 4c, Table 1). Group Green displays the highest decrease rate of −1.37 kg m^−2^ m^−1^ due to (a) abundant moisture to begin with and (b) a wide range of elevation (300–4000 m). Group Purple is associated with a relatively small but positive and statistically significant increase rate of 0.13 kg m^−2^ m^−1^. The ERA-Int cells located in the Qaidam desert basin (Group Yellow) and above 4000 m (Group Blue) correspond to very low TCWV (Fig. 4c). In contrast, CAPE increases with elevation for all groups and statistically significant increase rates are found for Groups Red, Purple and Blue (Fig. 4d and Table 1). Groups Green and Yellow exhibit rather small increase rates of CAPE. LCL decreases with elevation up to 4000 m and beyond that LCL either flattens out (Groups Red and Purple) or does not show a linear fit (Group Blue) (Fig. 4e). The decrease rates of LCL for Groups Red, Purple and Green are all statistically significant although Group Green displays the smallest rate of −0.05 m m^−1^ (Table 1). It appears that 4000 m is a pivotal height for sharp changes in moisture and LCL based on the ERA-Int reanalysis.

MERRA2 presents a largely consistent pattern with ERA-Int in terms of TCWV vertical gradient signs and magnitudes for all groups except for Group Blue for which MERRA2 shows a much larger negative TCWV gradient than ERA-Int (Table 1, Figs 5c and 4c). Consistency is also striking for LCL between MERRA2 and ERA-Int (Table 1, Figs 5e and 4e), except for Group Blue. For CAPE, MERRA2 presents statistically significant positive gradients for Groups Red and Purple as in ERA-Int although the magnitudes are much smaller for MERRA2 (Table 1). For Group Green, MERRA2 exhibits a statistically significant negative gradient of CAPE while ERA-Int shows a positive gradient. Also, MERRA2 displays zero gradients for Groups Yellow and Blue while ERA-Int shows positive gradients (Table 1). As will be explained in the Data and Method section, it appears that MERRA2’s CAPE computed using the 6-hourly model data is under-estimated when compared to ERA-Int’s CAPE computed using the hourly model data. However, the CAPE patterns for MERRA2 appear to be reasonable judging from the strong consistency between MERRA2 and ERA-Int in the gradients of precipitation, TCWV, LCL, and CAPE for Groups Red, Purple and to a lesser extent Yellow.

The above analyses based on ERA-Int and MERRA2 suggest that the observed positive gradients of precipitation for Groups Red and Yellow is due to the combined effects of negative LCL gradients and positive CAPE gradients that overwhelm those of negative TCWV gradients. For Group Purple, positive TCWV and CAPE gradients combined with a negative LCL gradient result in a large positive gradient of precipitation. For Group Green, ERA-Int suggests that the combined effects of a small negative LCL gradient and a small positive CAPE gradient may just balance out those of a negative TCWV gradient which gives rise to little change of precipitation with elevation for this group. For Group Blue, MERRA2 indicates that the observed negative gradient of precipitation is due to the combined effects of increasing LCL and decreasing TCWV with elevation.

Horizontally, both ERA-Int and MERRA2 reveal that during the wet season large precipitation over the southeastern TPS (Group Green) is due to the combined effects of abundant moisture, high CAPE and low LCL while the opposite is true for the Qaidam Basin (Group Yellow). This clear contrast is related to the prevailing atmospheric circulations in the different areas in the wet season. Group Green is located in the southeastern TPS that is affected primarily by the South and East Asia monsoons and not only TCWV but also CAPE and LCL favor precipitation. On the other hand, Group Yellow is located in a comparably low basin in the northwestern TPS surrounded on all sides by mountain ranges and is affected mainly by the moisture depleted westerlies and less so by the monsoons, which result in low CAPE and TCWV but high LCL, all detrimental to the development of precipitation there. For Groups Red and Purple in the same elevation range, more moisture, higher CAPE and lower LCL lead to more precipitation for the former than for the latter. In the interior of the TPS (Group Blue), moisture is low but CAPE is relatively high and LCL is relatively low, giving rise to moderate precipitation. Thus, largely consistent with the southeast-northwest horizontal gradient of precipitation shown in Fig. 2b, both TCWV and CAPE decrease but LCL increases from the southeast to the northwest of the TPS (Figs 4 and 5, Supplementary S6 and S7).

To determine the relative importance of the three variables in affecting the wet season precipitation, the standardized partial regression coefficients (SPRCs) as derived through multiple linear regression are investigated in Table 2. Due to the potential issue with CAPE magnitude for MERRA2 as mentioned before, the results for MERRA2 are provided here for reference purposes only. For ERA-Int, SPRCs for CAPE and LCL are statistically significant for all groups. But for TCWV only Group Red shows statistically significant SPRC, and SPRC is zero for Group Blue. SPRCs for CAPE are always larger in magnitude than those for LCL except for Groups Yellow and Blue for which the opposite is true. For MERRA2, SPRCs are generally consistent in sign with those for ERA-Int except for TCWV for Groups Green and Blue and CAPE for Groups Purple, Green and Blue. SPRCs for CAPE are generally much smaller in magnitude for MERRA2 than for ERA-Int probably related to the potential issue with MERRA2’s CAPE. If we focus more on ERA-Int, the analysis shows that CAPE and LCL are the most important variables that are responsible for the regional distinctive wet season precipitation gradients on the TPS and that TCWV appears to play a secondary role.

**Table 2:** Standardized partial regression coefficients^a^ of wet season precipitation Prcp with wet season TCWV, CAPE and LCL at the ERA-Int cells/MERRA2 cells.

	TCWV	CAPE	LCL
Red	0.16*/0.17*	0.70*/0.30*	−0.29*/−0.59*
Yellow	0.96/0.85*	1.00*/0.23	−1.54*/−1.82*
Purple	0.11/0.18	0.60*/−0.14	−0.33*/−0.91*
Green	0.12/−0.23	0.54*/0.21	−0.44*/−0.72*
Blue	0.00/0.39*	0.49*/−0.30*	−0.54*/−0.49*

*represents statistically significant values at 90% confidence level.

^a^Standardized partial regression coefficient is computed as: Partial regression coefficient b1 (b2, b3) × standard deviation of TCWV (CAPE, LCL) ÷ standard deviation of Prcp given Prcp = b0 + b1 × TCWV + b2 × CAPE + b3 × LCL.

In summary, four key points can be obtained: 1) wet season precipitation decreases with elevation in the interior TPS with terrain height greater than 4000 m, displays little change over the southeastern TPS, but increases elsewhere; 2) the increase of precipitation with elevation is the result of increasing CAPE and decreasing LCL with elevation that overwhelm the effects of decreasing TCWV with elevation; 3) the decrease of precipitation with elevation is due to the combined effects of increasing LCL and decreasing TCWV; and 4) little change of precipitation with elevation is caused by the combined effects of slightly decreasing LCL and slightly increasing CAPE that oppose decreasing TCWV.

## Discussion

On the TPS, wet season precipitation accounts for more than 70% of annual total precipitation at most stations. Horizontally, precipitation decreases from the southeast to the northwest as a result of the prevailing atmospheric circulations combined with the effects of local elevation and terrain. The stations are divided into five groups based on the vertical gradients of wet season precipitation. Both ERA-Int and MERRA2 resolve the observed positive gradients for Groups Red, Purple and Yellow but ERA-Int fails to represent the observed negative gradient for Group Blue while MERRA2 fails to represent the observed negligible gradient for Group Green. CAPE, LCL and TCWV from ERA-Int and MERRA2 explain the observed distinctive regional patterns of the wet season precipitation gradients reasonably well.

Precipitation changes with elevation have been studied over the Karakoram-Hindukush-western Himalayas (KHH) region^26, 27^ as well as over the Tibetan Plateau^28^. Some of these studies also reveal spatially varying vertical gradients of precipitation, more or less consistent with the findings in this study; however, these studies do not examine the mechanisms behind the spatial variations as is done in this study. This study relates the spatial variations in vertical gradients of precipitation to those of TCWV, LCL and CAPE, which could further spur the interests for investigating in detail the atmospheric circulations and climate systems that give rise to the patterns. Such studies could have practical implications for long-term forecasts and investigations of future changes under global warming. Issues revealed in this study include: 1) the scarcity of observations for areas higher than 4000 m due to technical and accessibility problems^27^ greatly limit the number of stations and reanalysis cells for those areas; and 2) differing performance of the global reanalysis products may point to the need of improved horizontal resolution and model physics for complex topography over the TPS, especially in southeast TPS (Group Green) and interior TPS above 4000 m (Group Blue). More high-altitude meteorological stations could lead to a better understanding of the spatial variations of vertical precipitation profiles. How to overcome technical difficulties for acquiring long-term quality *in situ* observations at high elevations indeed is an urgent issue.

## Data and Method

We used the daily precipitation and station meta data downloaded from the China Meteorological Bureau’s Data Sharing Website (https://data.cma.cn), and can be downloaded from http://en.tpedatabase.cn/portal/MetaDataInfo.jsp?MetaDataId=249472.

Using the daily data, we calculated the wet season (May–September) total precipitation for each year during 1979–2015 and the means. In total, 184 stations with at least 10 years of observations during 1979–2015 were selected for the analyses (Fig. 2a).

Through maximizing the linear regression coefficients (slopes) between wet season precipitation and elevation of station, 5 statistically significantly different coefficients at the 90% confidence level are identified among all stations. Hence, the stations are divided into 5 groups that have different regression coefficients. This division reflects the influence of both atmospheric circulations that prevail over different regions and complex topography on wet season precipitation.

ERA Interim (ERA-Int) monthly precipitation, total column water vapor (TCWV), convective available potential energy (CAPE), 2 m air temperature (T_air_) and dew point temperature (T_dew_) for 1979–2015 at 0.75° × 0.75° resolution were downloaded from the ECMWF website (https://apps.ecmwf.int/datasets/data/interim-full-daily/levtype=sfc/, accessed on August 1, 2016). The second generation MERRA (MERRA2) monthly precipitation, TCWV, 2 m air temperature (T_air_) and dew point temperature (T_dew_) for 1980–2015 at 0.5° × 0.67° resolution were downloaded from the MERRA2 website (https://disc.gsfc.nasa.gov/uui/datasets?keywords=%22MERRA-2%22, accessed on February 3, 2017). MERRA2’s CAPE was calculated by using MERAA2’s 6-hourly data for pressure, temperature, mixing ratio, and height at surface and at model levels using NCAR Command Language (NCL) software. Lifting condensation level (LCL) for both ERA-Int and MERRA2 was calculated using the Espy’s equation as follows:

1 $${\rm{LCL}}=125\times ({{\rm{T}}}_{{\rm{air}}}-{{\rm{T}}}_{{\rm{dew}}})$$

where LCL is in meters, T_air_ and T_dew_ are in °C.

MERRA2 has a slightly finer resolution than ERA-Int. MERRA2’s CAPE is the monthly mean of daily 6-hourly (i.e., at 00, 06, 12 and 18 UTC) values while ERA-Int CAPE is the monthly mean of daily hourly values. 00, 06, 12 and 18 UTC correspond to 08 AM, 02 PM, 08 PM and 02 AM Local Standard Time (LST) over the Tibetan Plateau. Considering the fact that during the warm season (Mary–September), CAPE is much larger during late morning and early afternoon hours than during the nighttime hours, it is reasonable to believe that MERRA2’s CAPE is significantly under-estimated compared to ERA-Int CAPE.

Maps of the wet season total precipitation and TCWV, and wet season mean CAPE and LCL are shown in the supplementary material along with the ERA-Int elevation at 0.75° × 0.75° resolution (Figures S6, S7). The selection of the ERA-Int/MERRA2 cells for comparison with the station observations is based on the correlation coefficients (R) of the normalized annual wet season precipitation between the ERA-Int/MERRA2 cells and their corresponding stations: only those cells that display statistically significant R at 90% confidence level are chosen. The statistically significant R ranges from 0.22 to 0.84 for ERA-Int and from 0.23 to 0.80 for MERRA2. In total, 132 ERA-Int and 142 MERRA2 cells are selected (Table 1, Figs 4a, 5a). In the case when multiple stations are covered by a single ERA-Int/MERRA2 cell, we chose the station that has the longest observation record during 1979–2015 for the correlation analysis. Figures S8 and S9 in the supplementary material presents the normalized annual wet season precipitation time series at the ERA-Int/MERRA2 cells and the stations, which generally show reasonably consistent patterns in magnitude and evolution between the ERA-Int/MERRA2 reanalyses and observations. All statistical significance test used is the two tailed student t test with p < 0.10 as the statistical significance level.

## Electronic supplementary material

Supplementary Information
