# Supplementary material for: Spatial patterns of wet season precipitation vertical gradients on the Tibetan Plateau and the surroundings
Source: Sci Rep. 2017 Jul 11;7:5057. doi: 10.1038/s41598-017-05345-6 (PMC5506045; doi:10.1038/s41598-017-05345-6)
Supplement: Supplementary file 1 — Supplementary Information [file 41598_2017_5345_MOESM1_ESM.pdf]

Spatial patterns of wet season precipitation vertical gradients on the Tibetan Plateau and the surroundings

Lan Cuo[1,2,3] and Yongxin Zhang[4]

[1] Center for Excellence in Tibetan Plateau Earth Sciences, Beijing, China

[2] Key Laboratory of Tibetan Environment Changes and Land Surface Processes, Institute of Tibetan Plateau Research, Chinese Academy of Sciences, Beijing, China

[3] University of Chinese Academy of Sciences, Beijing, China

[4] Research Applications Laboratory and Climate and Global Dynamics Laboratory, National Center for Atmospheric Research, Boulder, Colorado, USA

Corresponding Author:

Lan Cuo

Building 3, Courtyard 16, Chaoyang District

Beijing, 100101, China

Email: lancuo@itpcas.ac.cn

Tel: 86-010-84097091

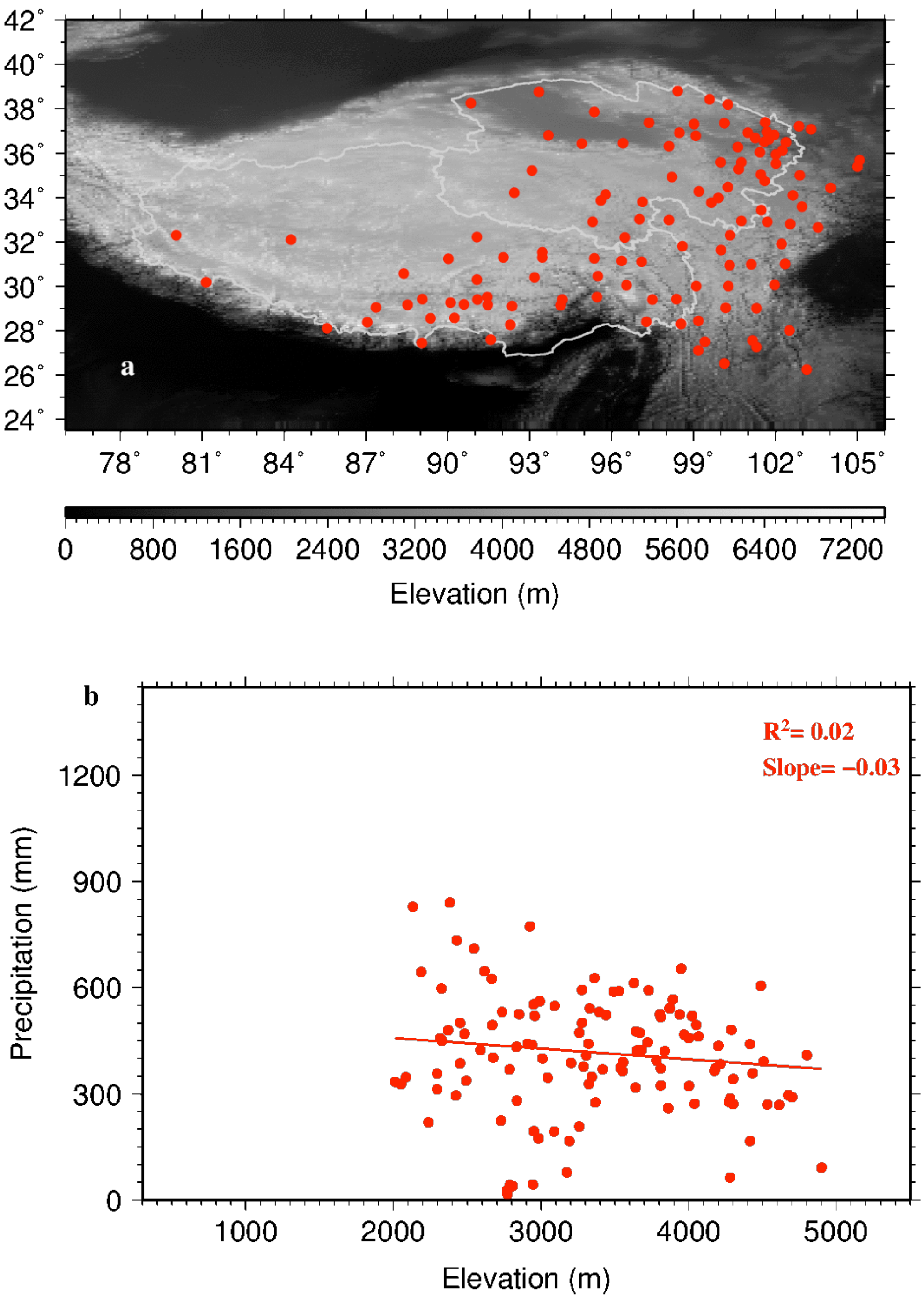

Figure S1 Locations of stations with elevation greater than 2000 m on the Tibetan Plateau and the surroundings (TPS), and their mean annual wet season precipitation change with elevation. This figure was plotted using the Generic Mapping Tools (GMT) V4.5.0 (URL: https://www.soest.hawaii.edu/gmt/).

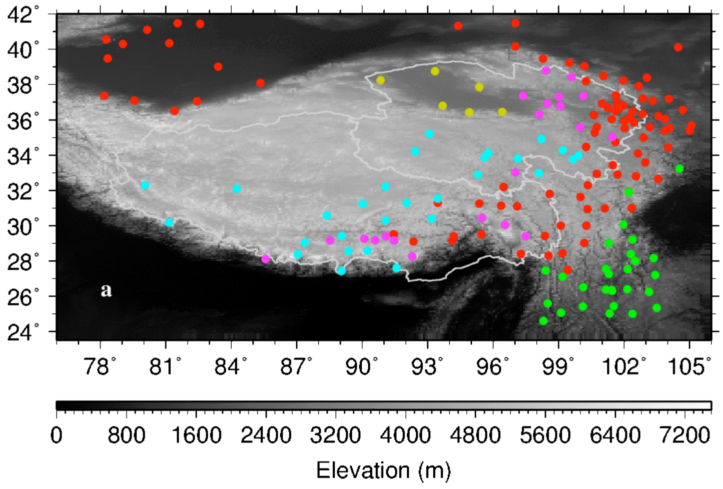

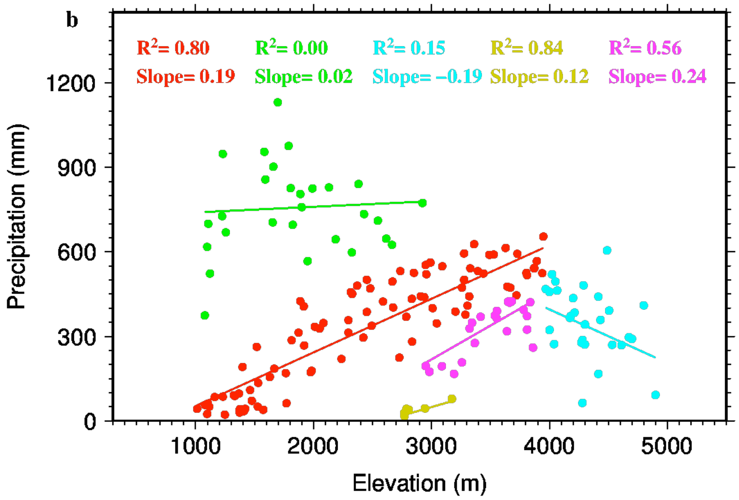

Figure S2 (a) Locations of 5 groups of stations that display different precipitation gradients with elevation identified in (b) on the TPS. Stations are all located above 1000 m above sea level. This figure was plotted using the Generic Mapping Tools (GMT) V4.5.0 (URL: https://www.soest.hawaii.edu/gmt/).

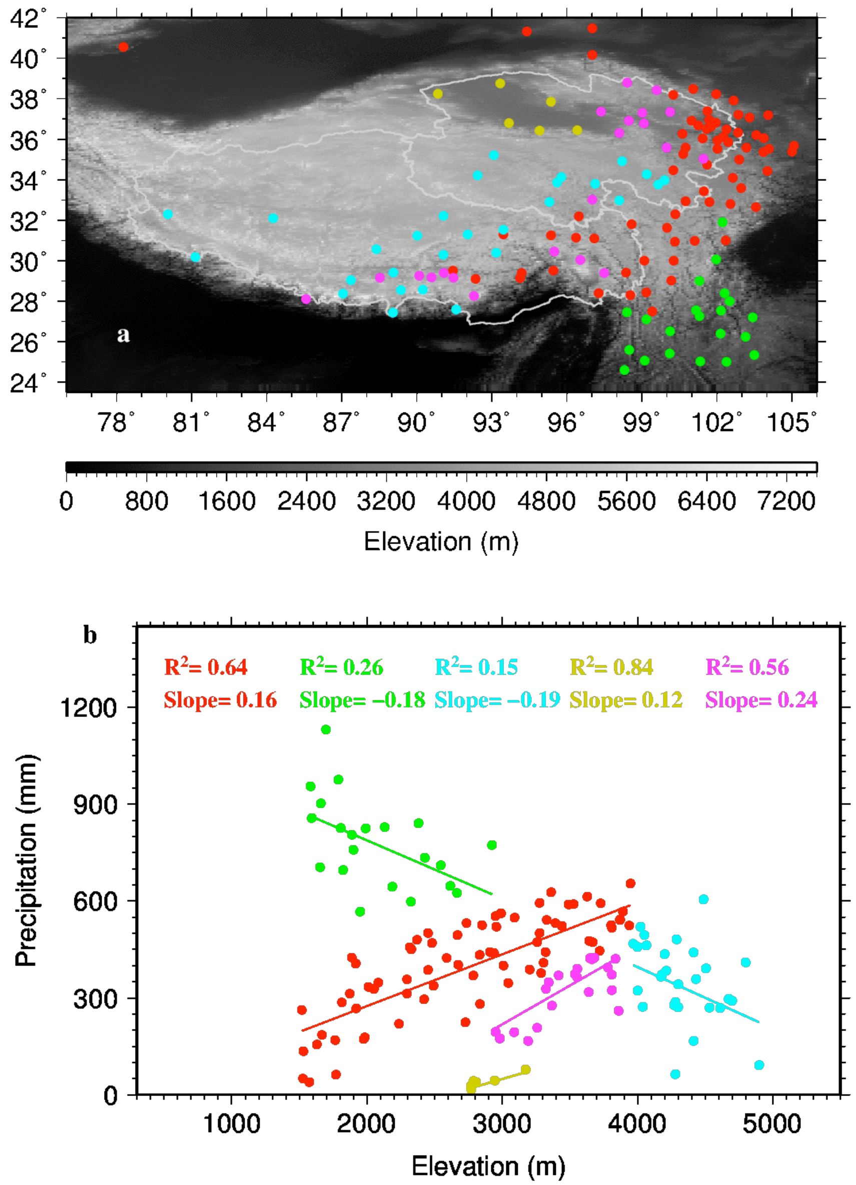

Figure S3 (a) Locations of 5 groups of stations that display different precipitation gradients with elevation identified in (b) on the TPS. Stations are all located above 1500 m above sea level. This figure was plotted using the Generic Mapping Tools (GMT) V4.5.0 (URL: https://www.soest.hawaii.edu/gmt/).

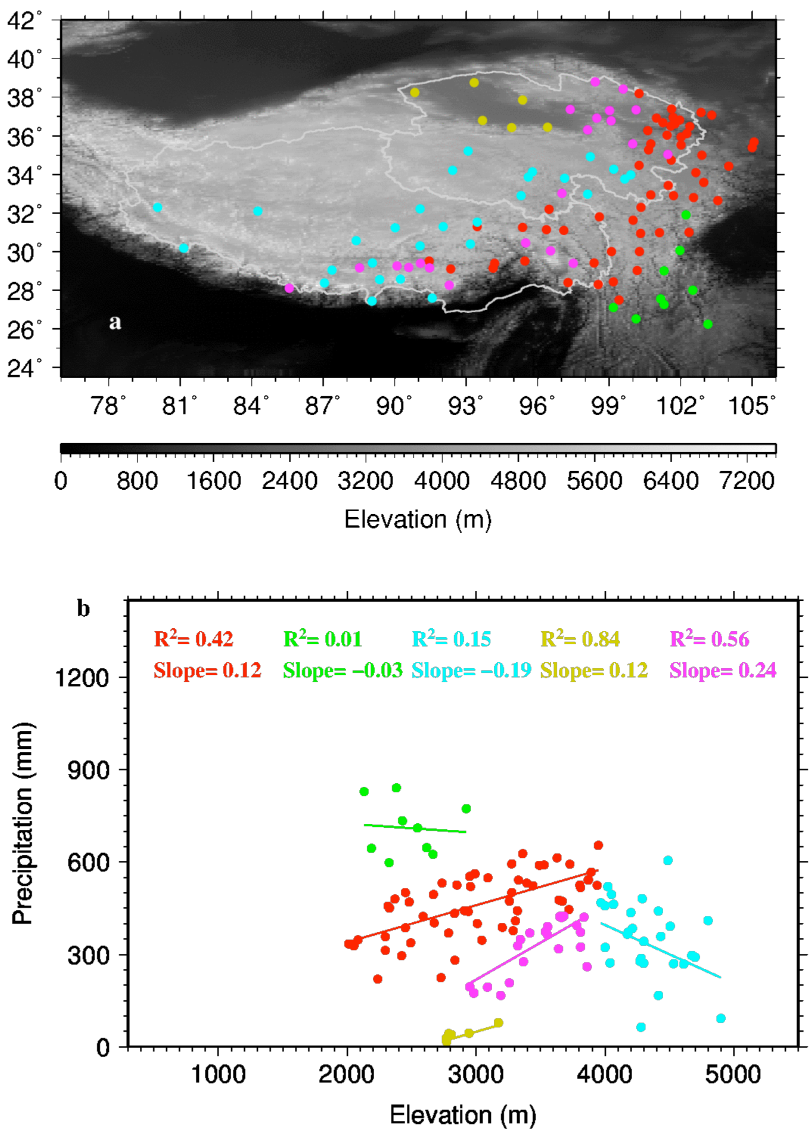

Figure S4 (a) Locations of 5 groups of stations that display different precipitation gradients with elevation identified in (b) on the TPS. Stations are all located above 2000 m above sea level. This figure was plotted using the Generic Mapping Tools (GMT) V4.5.0 (URL: https://www.soest.hawaii.edu/gmt/).

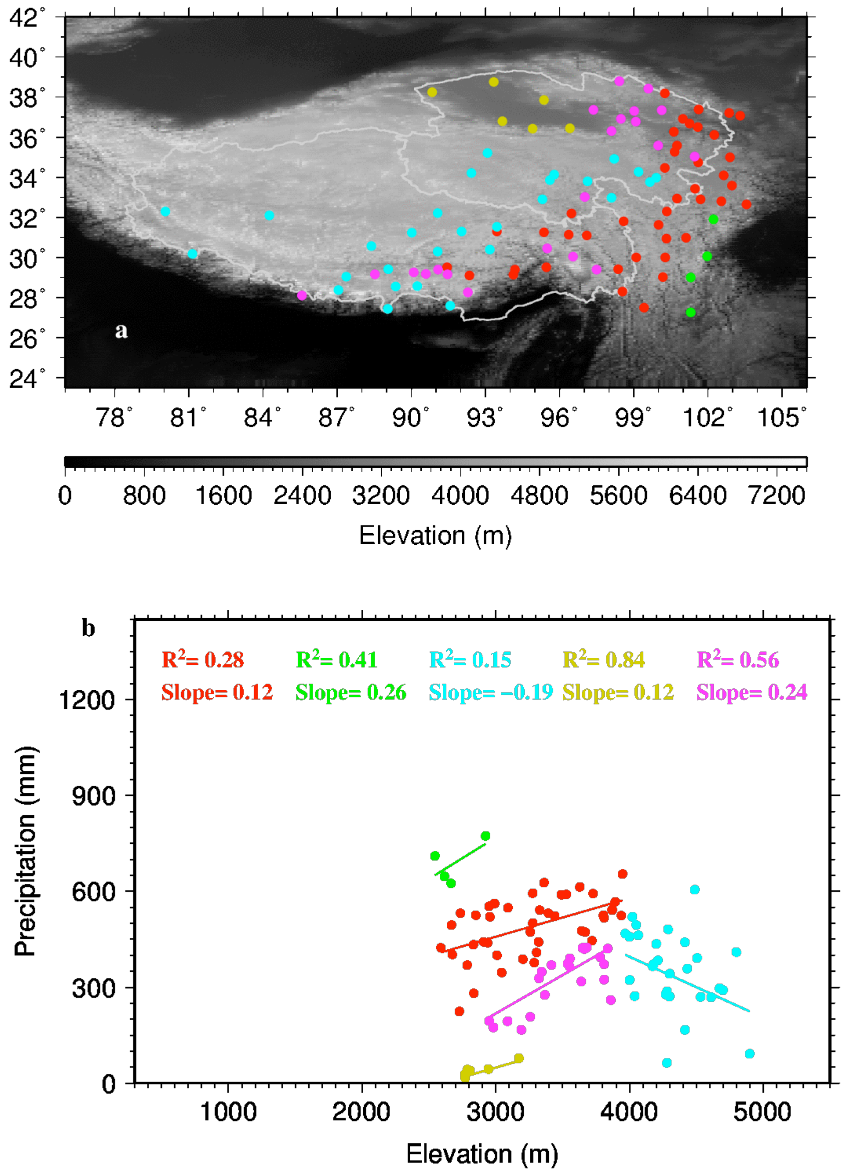

Figure S5 (a) Locations of 5 groups of stations that display different precipitation gradients with elevation identified in (b) on the TPS. Stations are all located above 2500 m above sea level. This figure was plotted using the Generic Mapping Tools (GMT) V4.5.0 (URL: https://www.soest.hawaii.edu/gmt/).

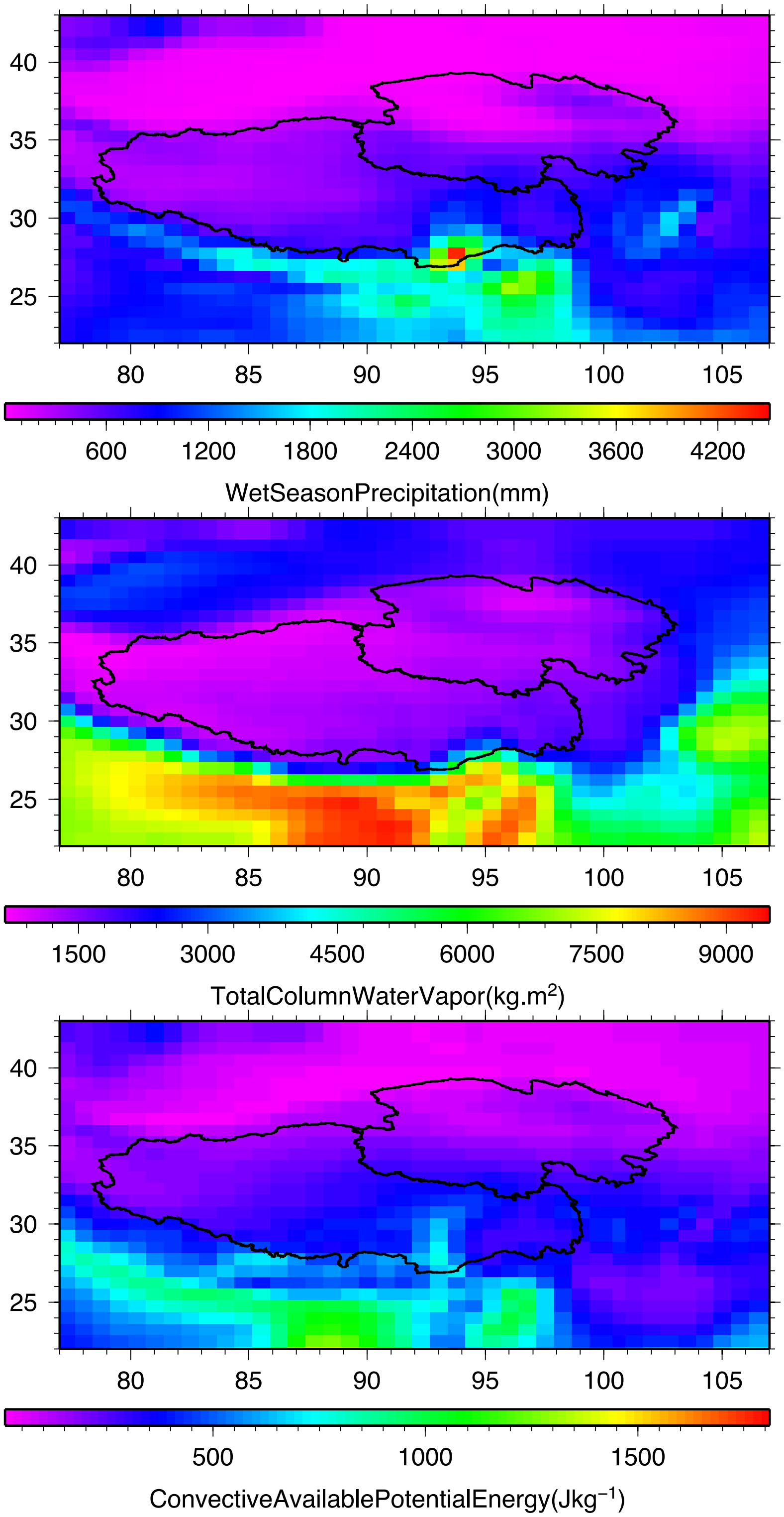

Figure S6. ERA-Interim mean wet season precipitation, total column water vapor and convective available potential energy over the TPS during 1979-2015. This figure was plotted using the Generic Mapping Tools (GMT) V4.5.0 (URL: https://www.soest.hawaii.edu/gmt/).

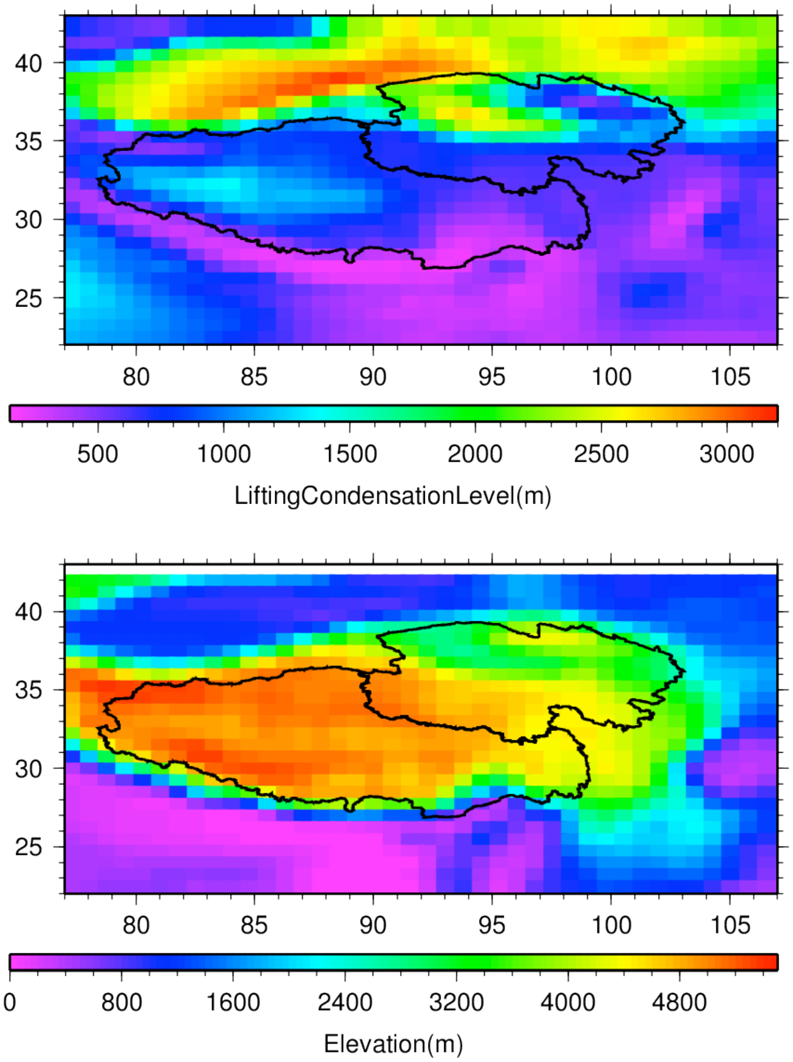

Figure S7. Lifting condensation level over the TPS derived from ERA-Interim 2-m air temperature and dew point temperature during 1979-2015 and ERA-Interim elevation. This figure was plotted using the Generic Mapping Tools (GMT) V4.5.0 (URL: https://www.soest.hawaii.edu/gmt/).

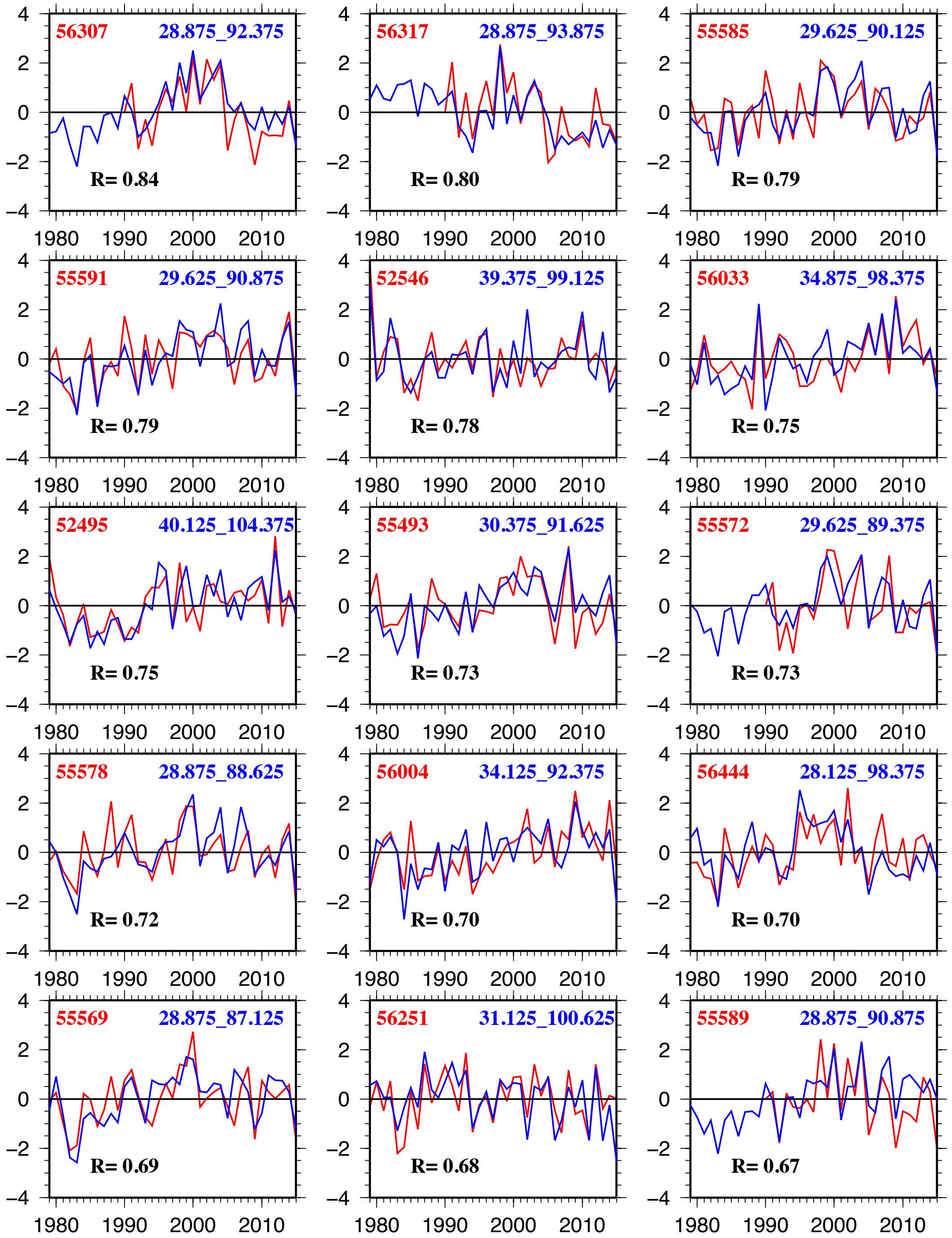

Figure S8. Standard deviation normalized wet season precipitation at the ERA-Interim cells and the corresponding stations. Station id is in red, and cell latitude and longitude are in blue. Correlation coefficient (R) is also shown. This figure was plotted using the Generic Mapping Tools (GMT) V4.5.0 (URL: https://www.soest.hawaii.edu/gmt/).

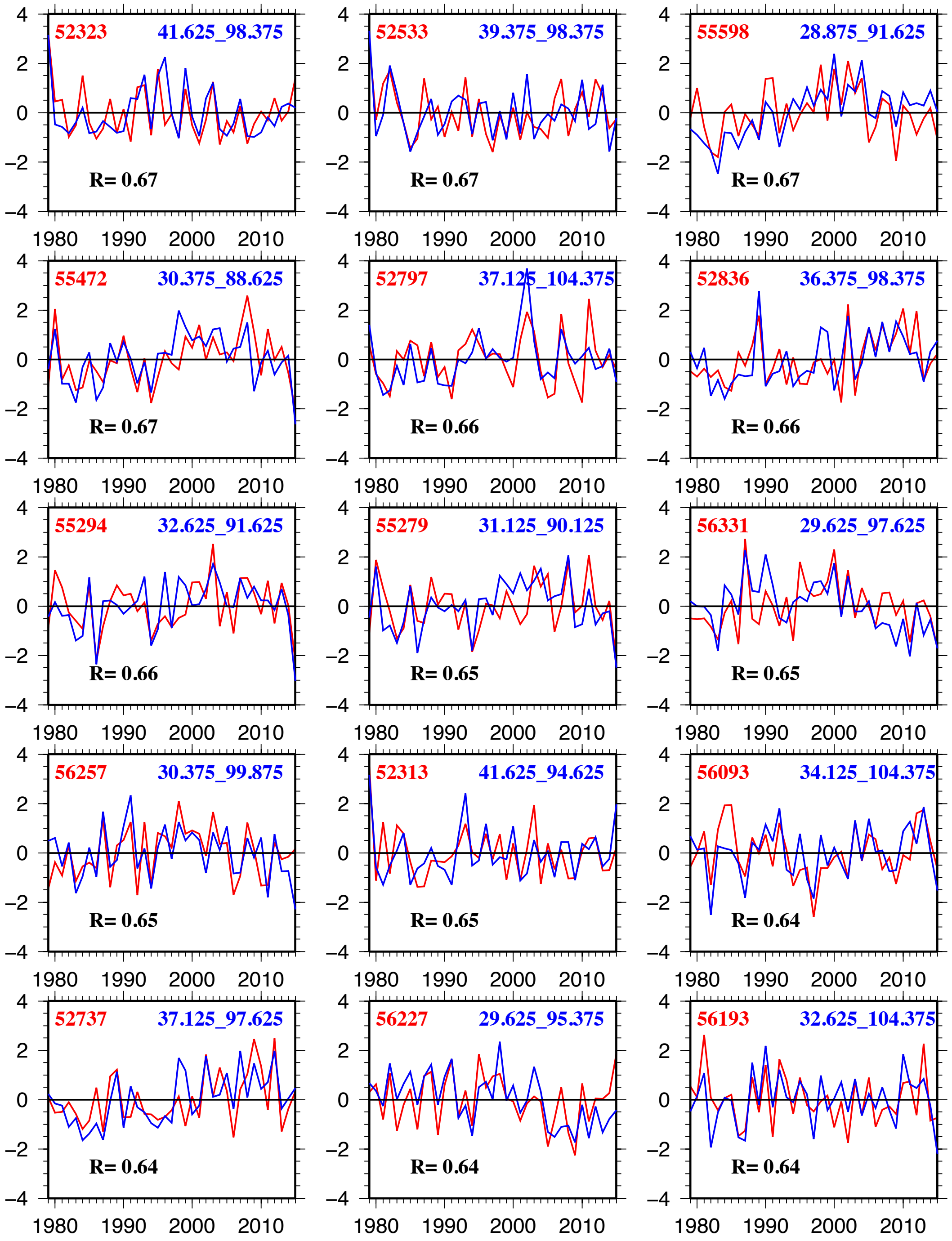

Figure S8. Continued. This figure was plotted using the Generic Mapping Tools (GMT) V4.5.0 (URL: https://www.soest.hawaii.edu/gmt/).

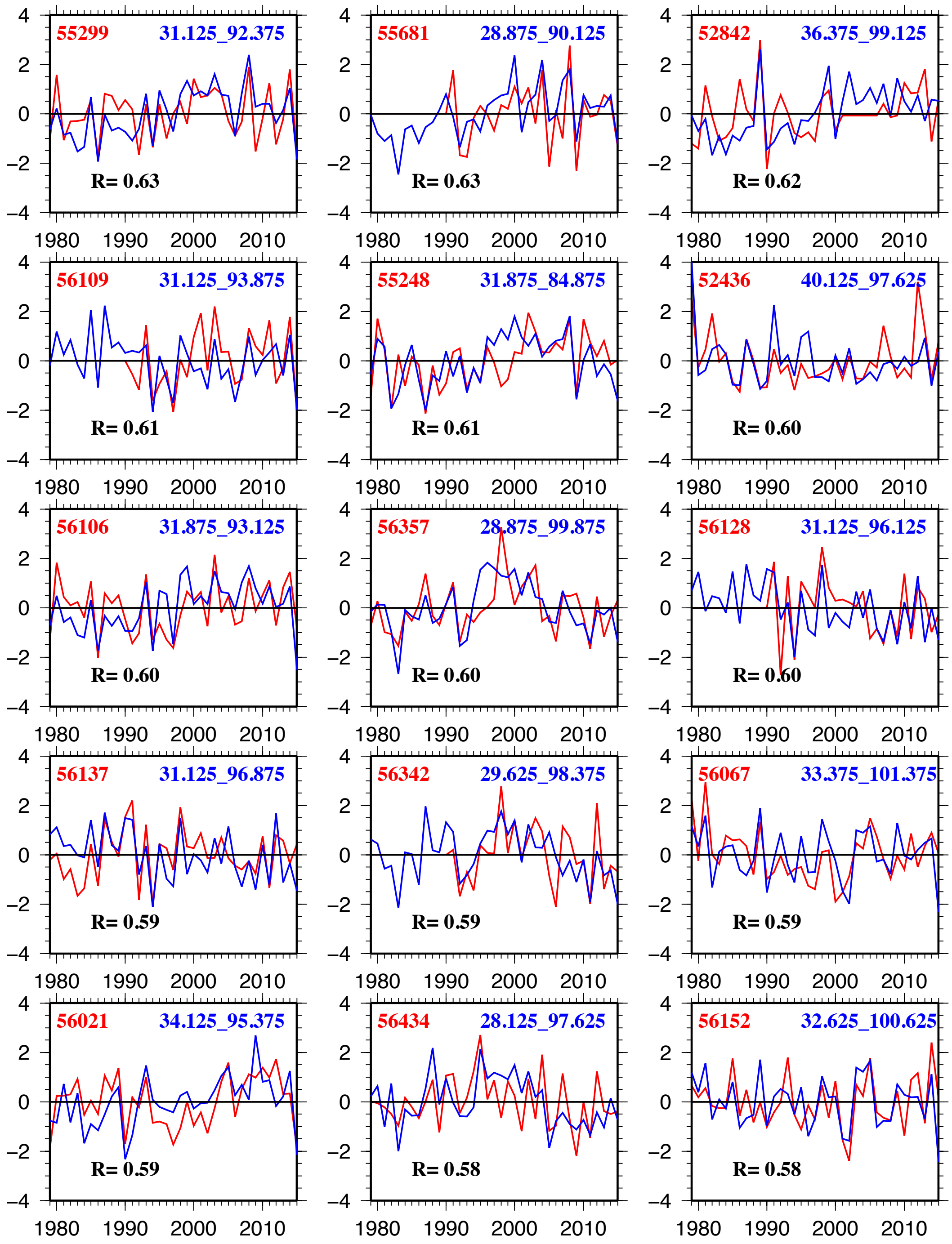

Figure S8. Continued. This figure was plotted using the Generic Mapping Tools (GMT) V4.5.0 (URL: https://www.soest.hawaii.edu/gmt/).

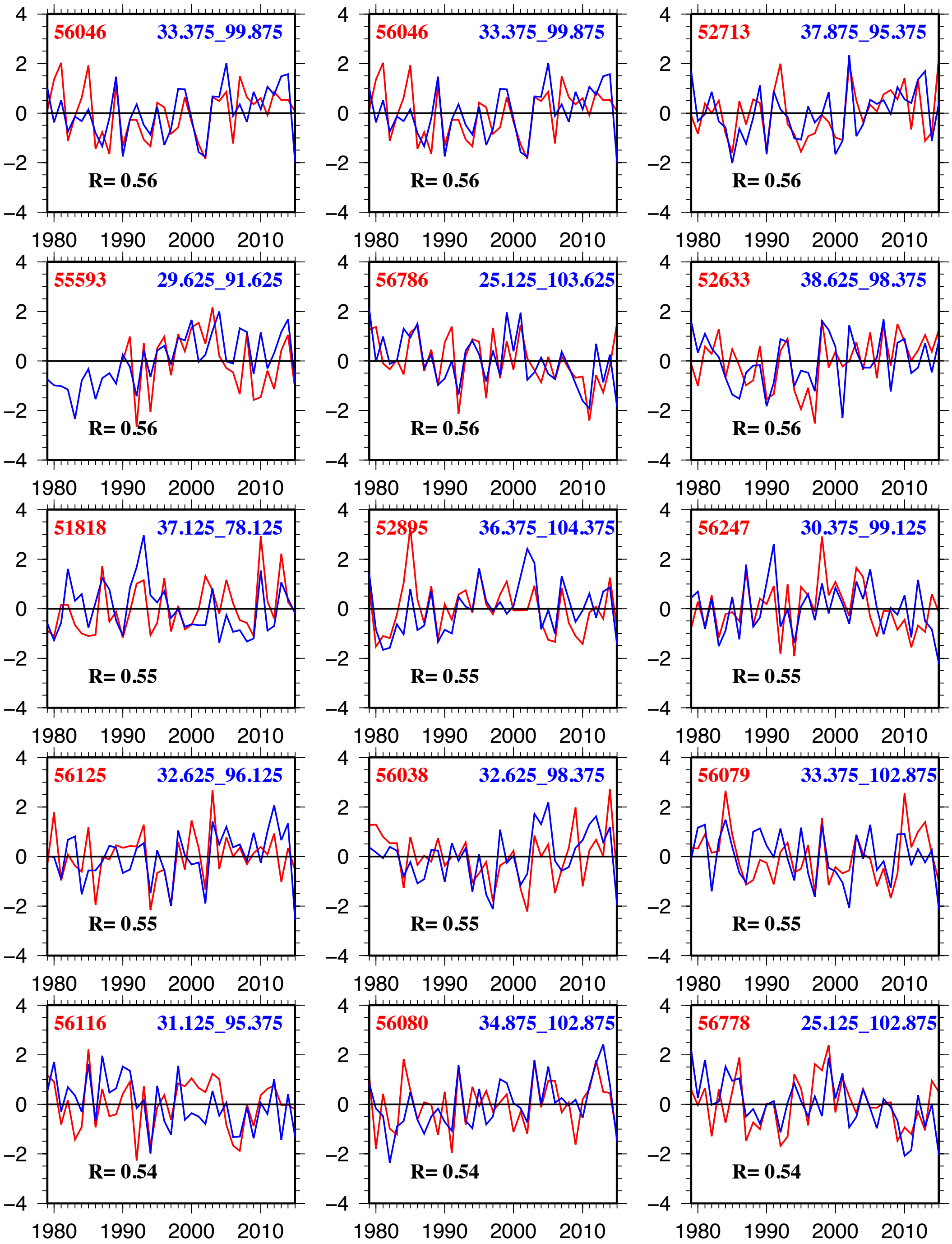

Figure S8. Continued. This figure was plotted using the Generic Mapping Tools (GMT) V4.5.0 (URL: https://www.soest.hawaii.edu/gmt/).

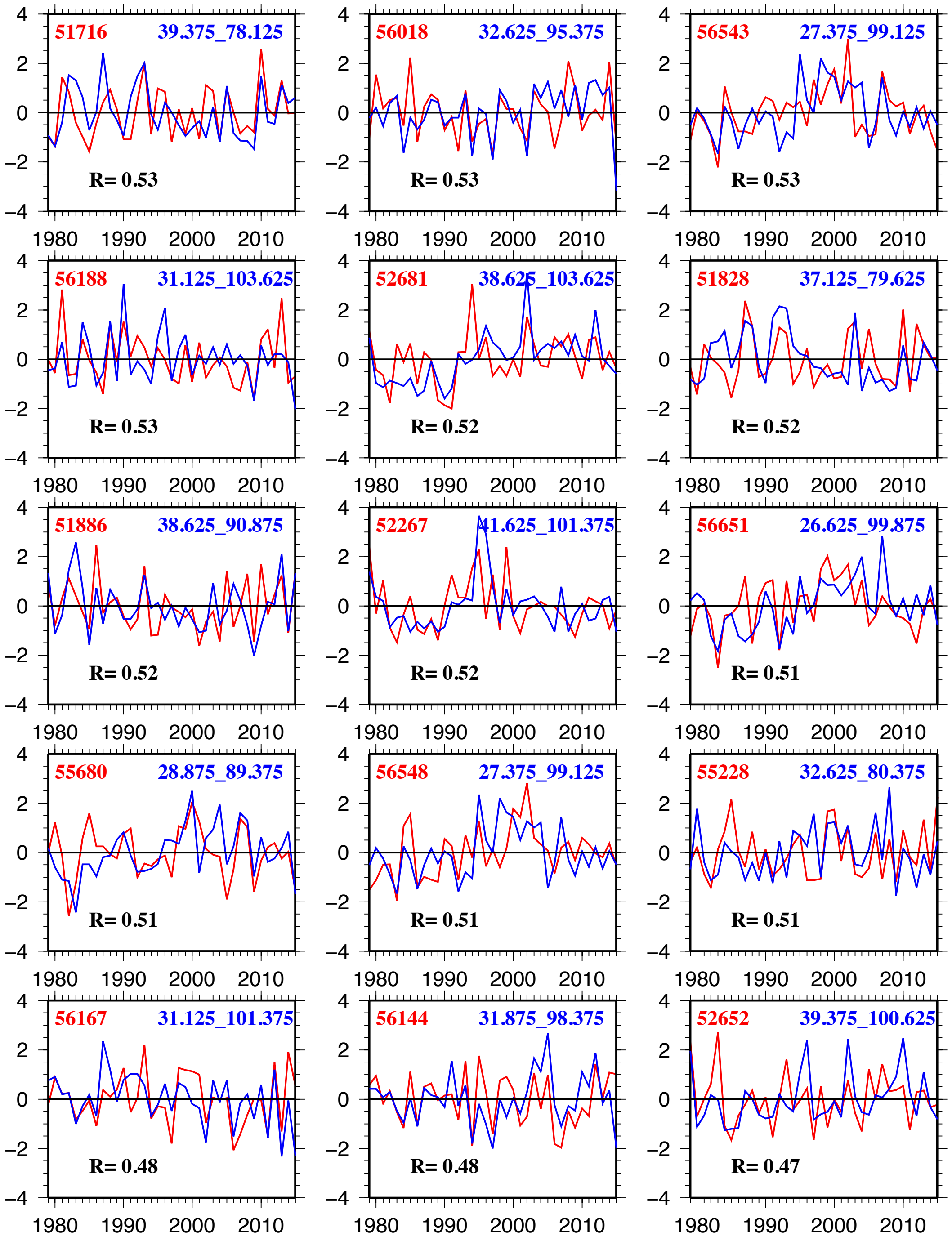

Figure S8. Continued. This figure was plotted using the Generic Mapping Tools (GMT) V4.5.0 (URL: https://www.soest.hawaii.edu/gmt/).

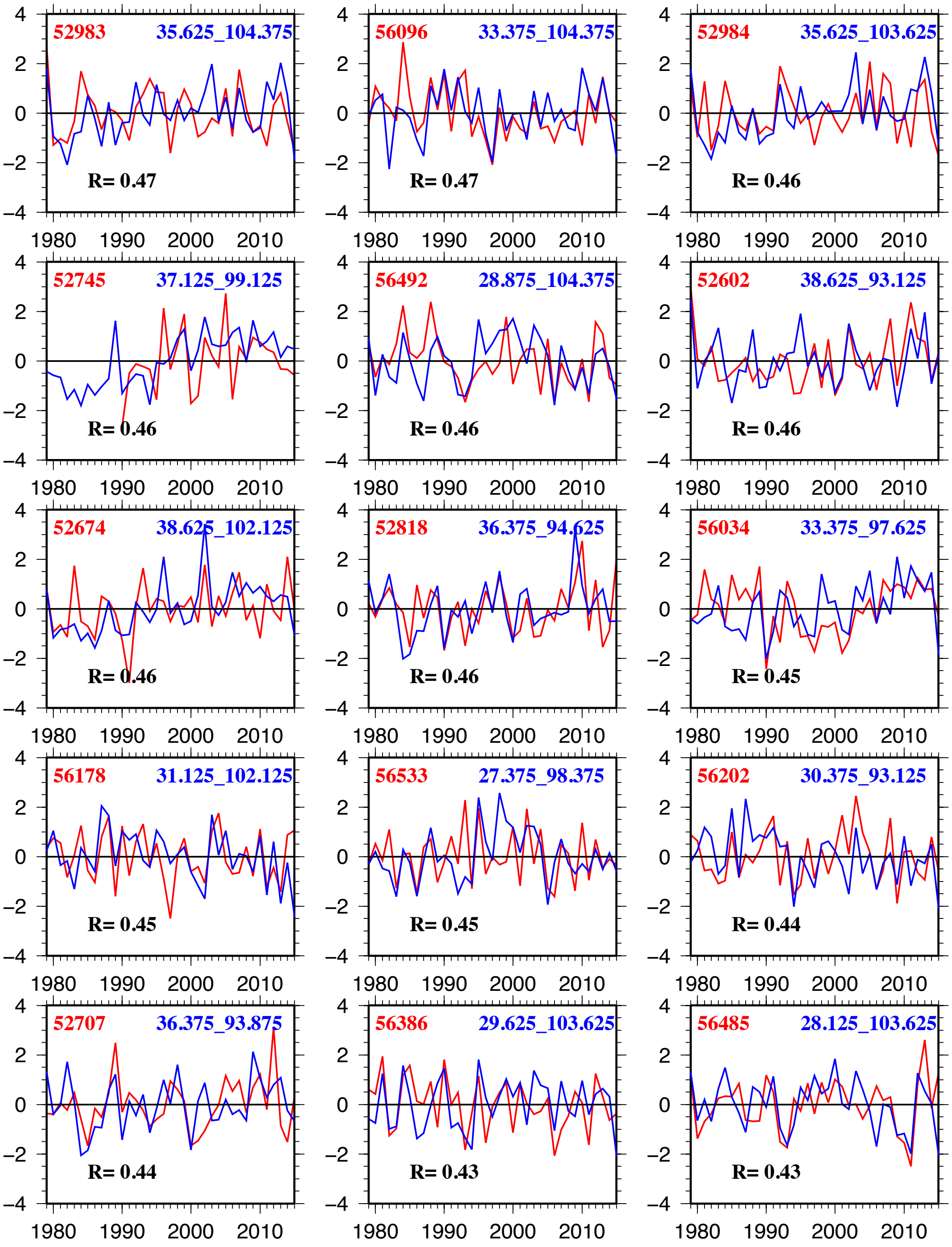

Figure S8. Continued. This figure was plotted using the Generic Mapping Tools (GMT) V4.5.0 (URL: https://www.soest.hawaii.edu/gmt/).

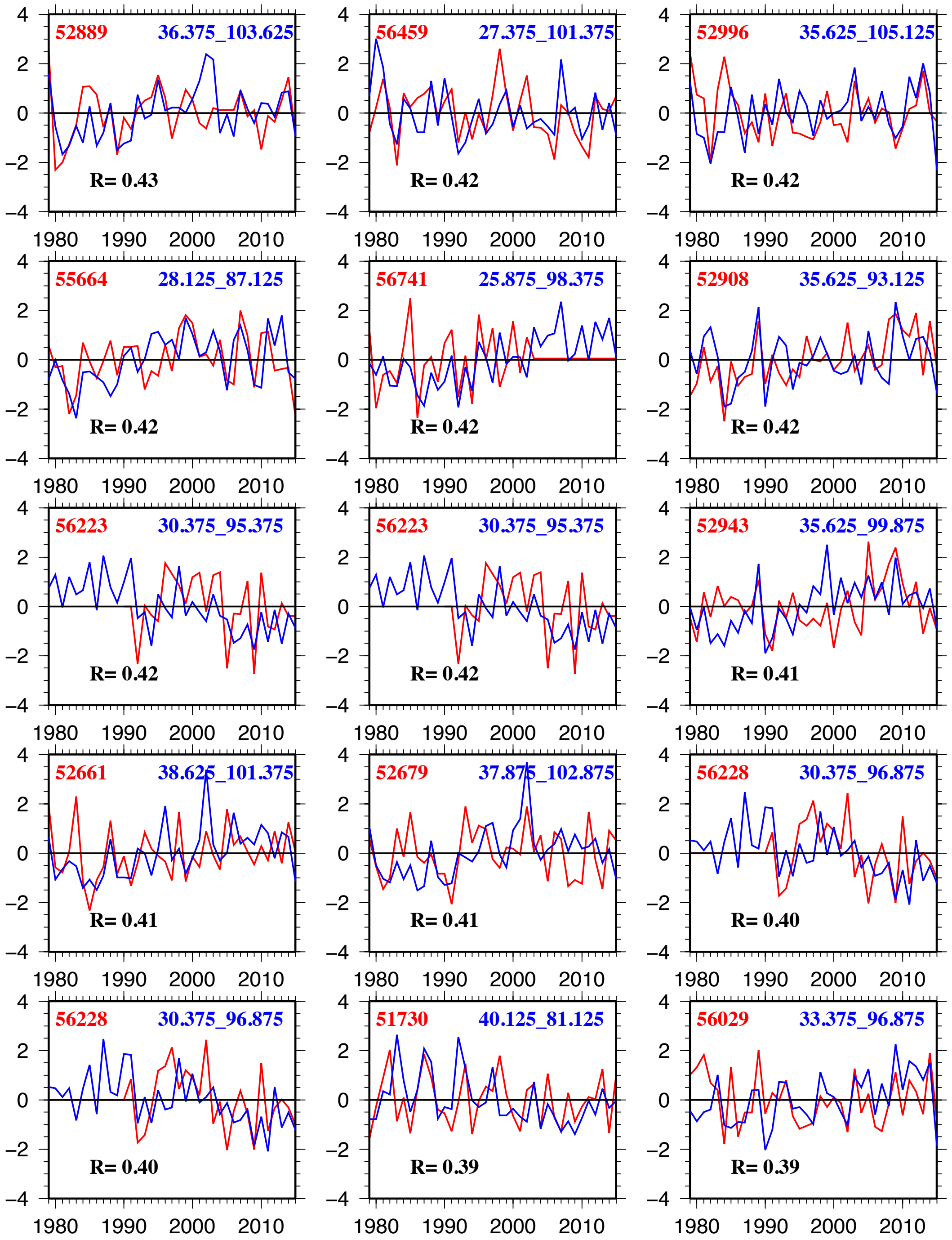

Figure S8. Continued. This figure was plotted using the Generic Mapping Tools (GMT) V4.5.0 (URL: https://www.soest.hawaii.edu/gmt/).

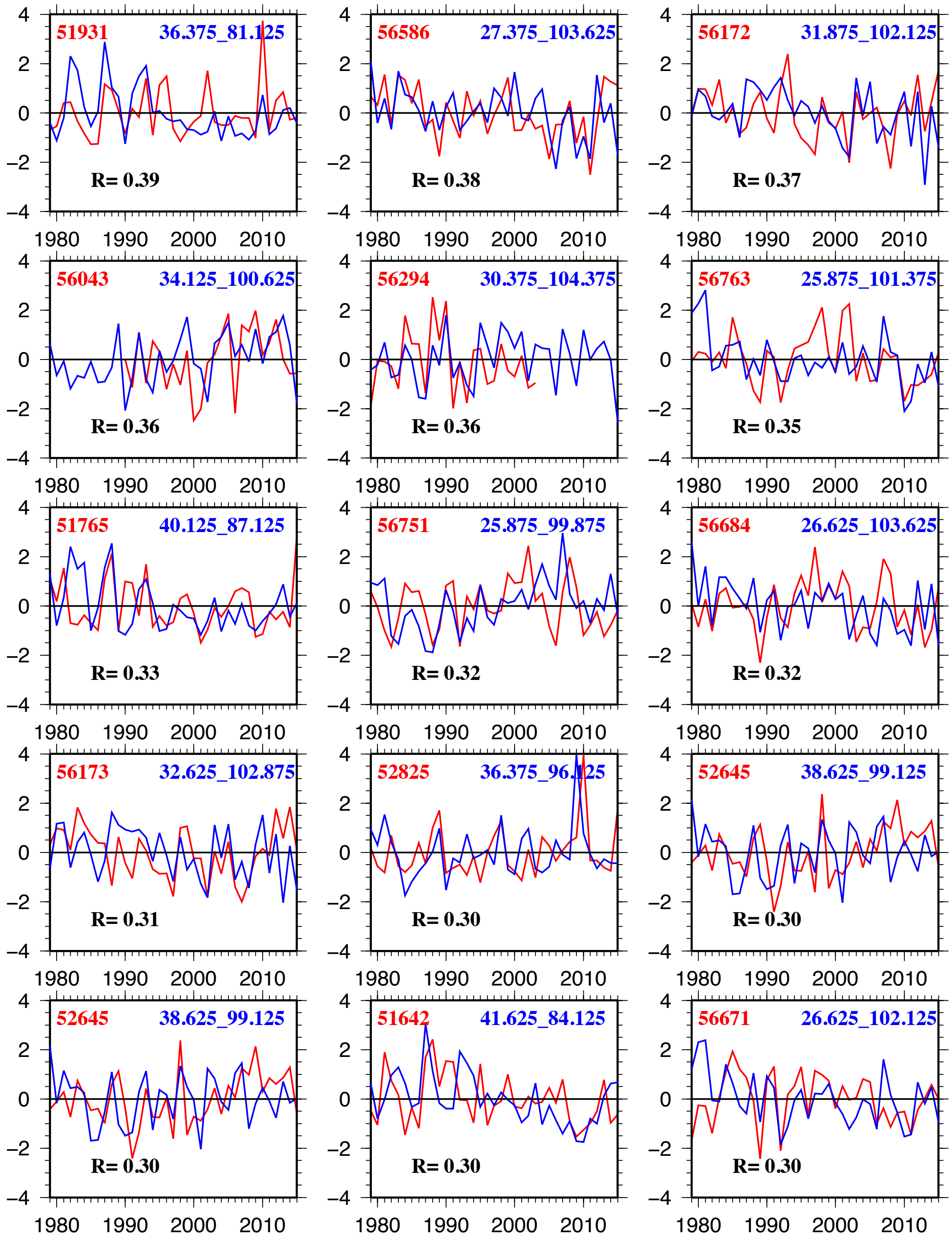

Figure S8. Continued. This figure was plotted using the Generic Mapping Tools (GMT) V4.5.0 (URL: https://www.soest.hawaii.edu/gmt/).

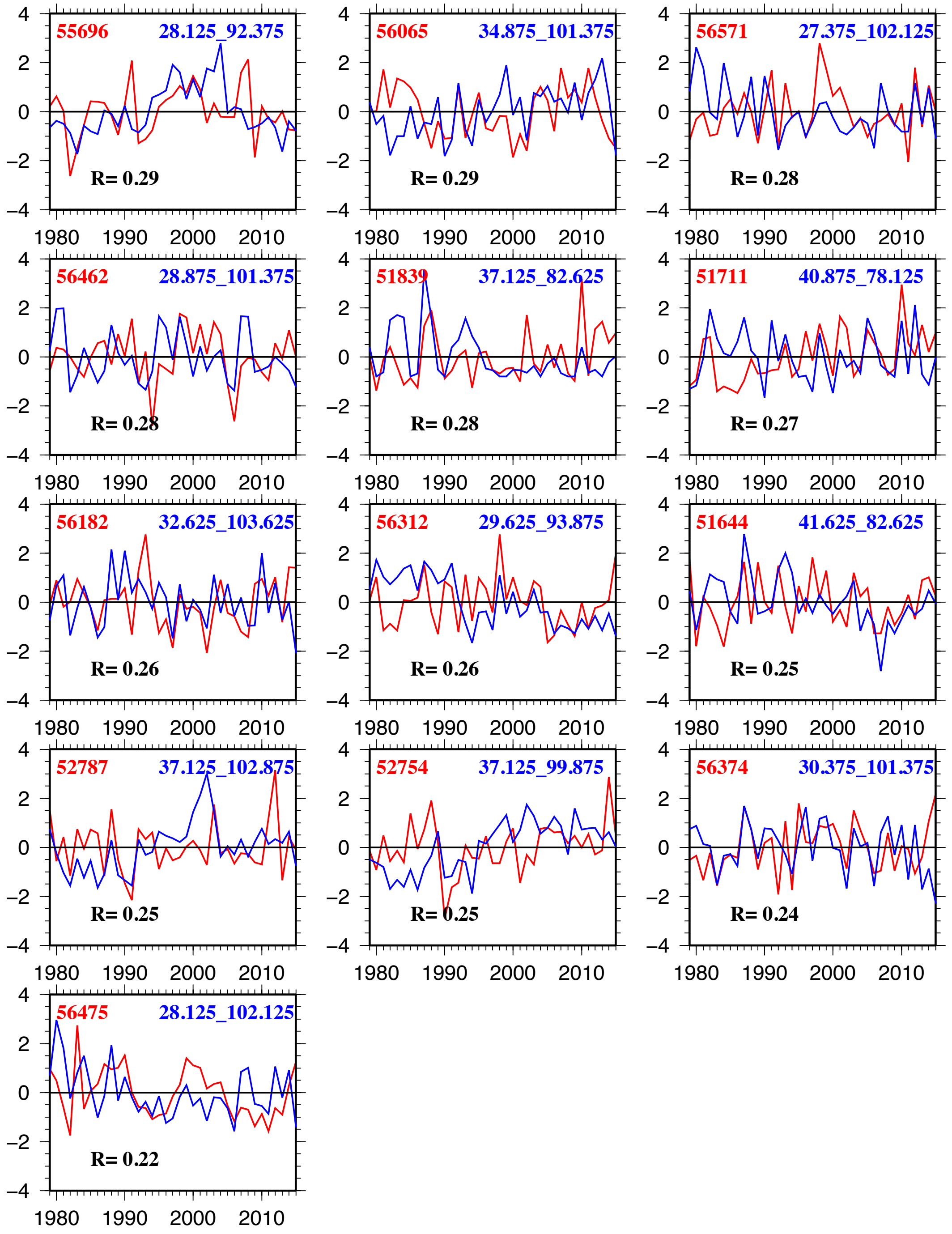

Figure S8. Continued. This figure was plotted using the Generic Mapping Tools (GMT) V4.5.0 (URL: https://www.soest.hawaii.edu/gmt/).

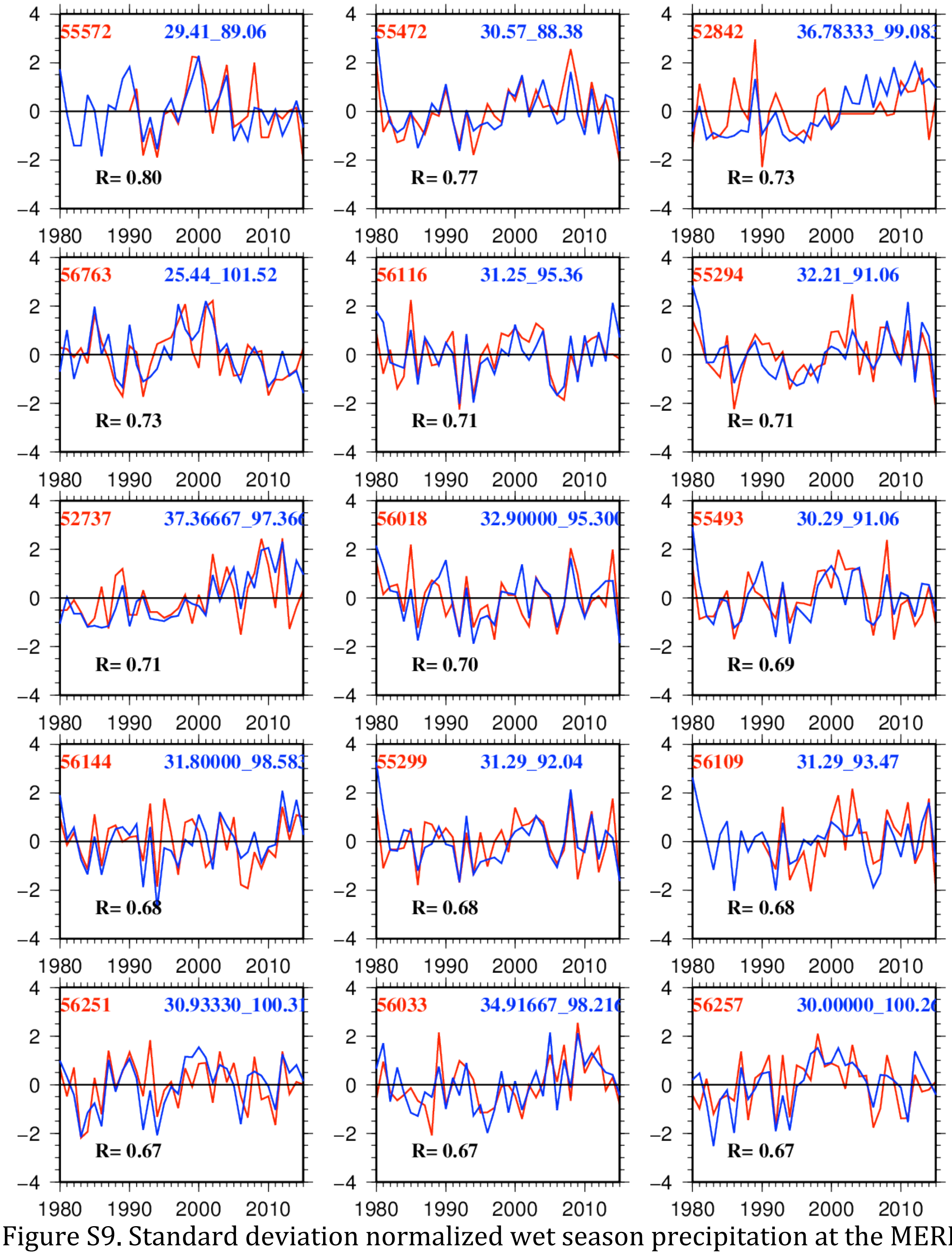

Figure S9. Standard deviation normalized wet season precipitation at the MERRA2 cells (blue line) and the corresponding stations (red lines). Station id is in red, and station latitude and longitude are in blue. Correlation coefficient (R) is also shown. This figure was plotted using the Generic Mapping Tools (GMT) V4.5.0 (URL: https://www.soest.hawaii.edu/gmt/).

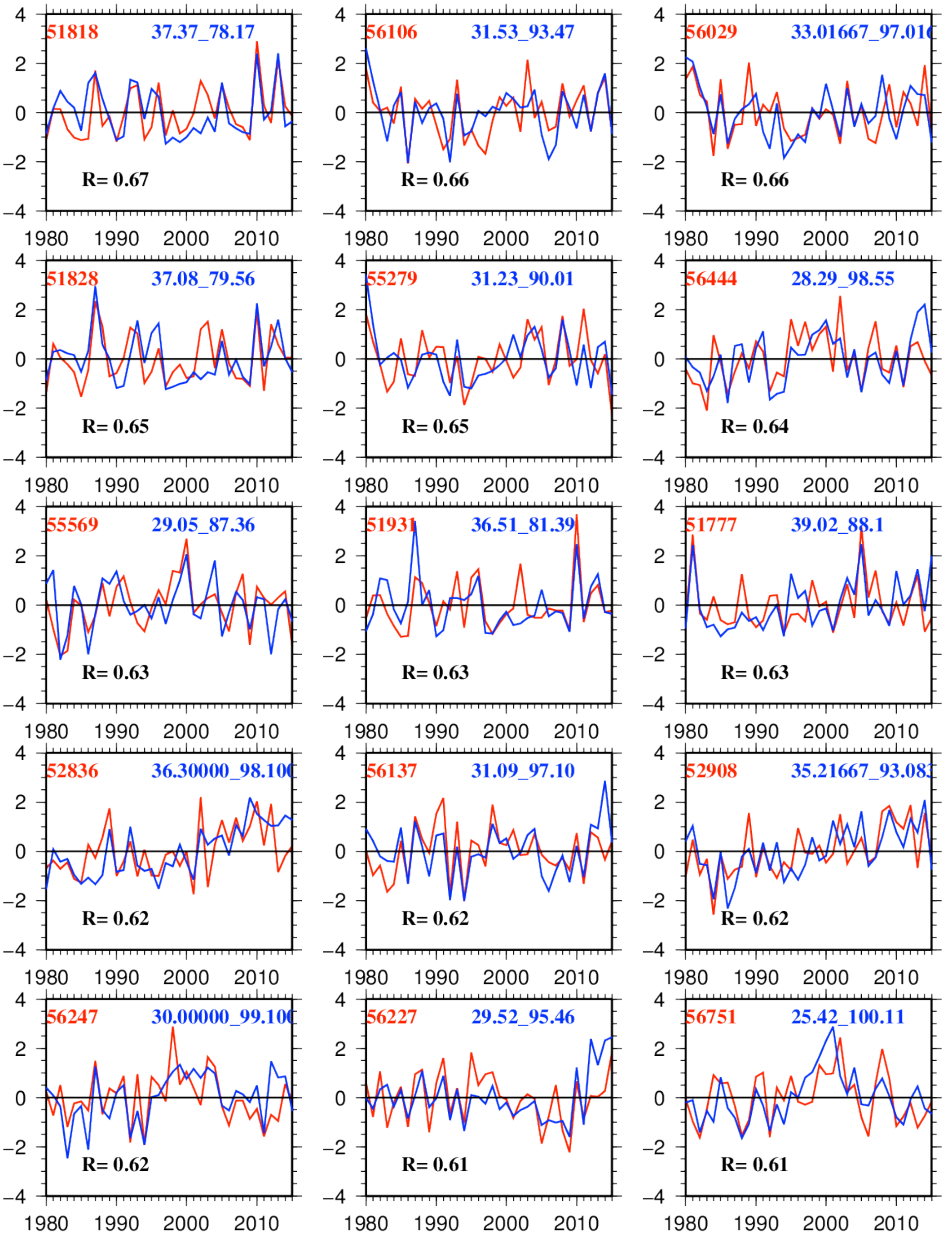

Figure S9. Continued. This figure was plotted using the Generic Mapping Tools (GMT) V4.5.0 (URL: https://www.soest.hawaii.edu/gmt/).

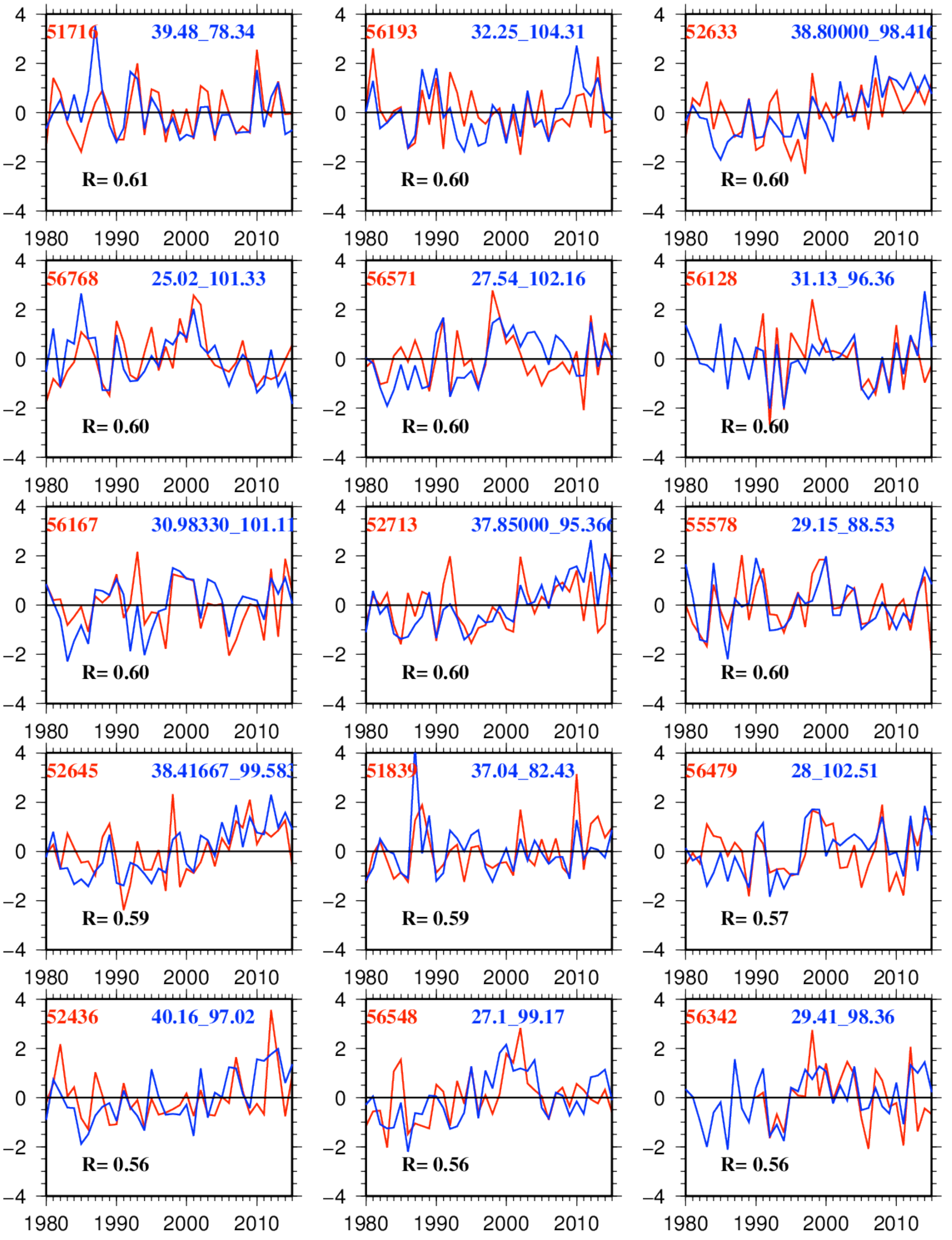

Figure S9. Continued. This figure was plotted using the Generic Mapping Tools (GMT) V4.5.0 (URL: https://www.soest.hawaii.edu/gmt/).

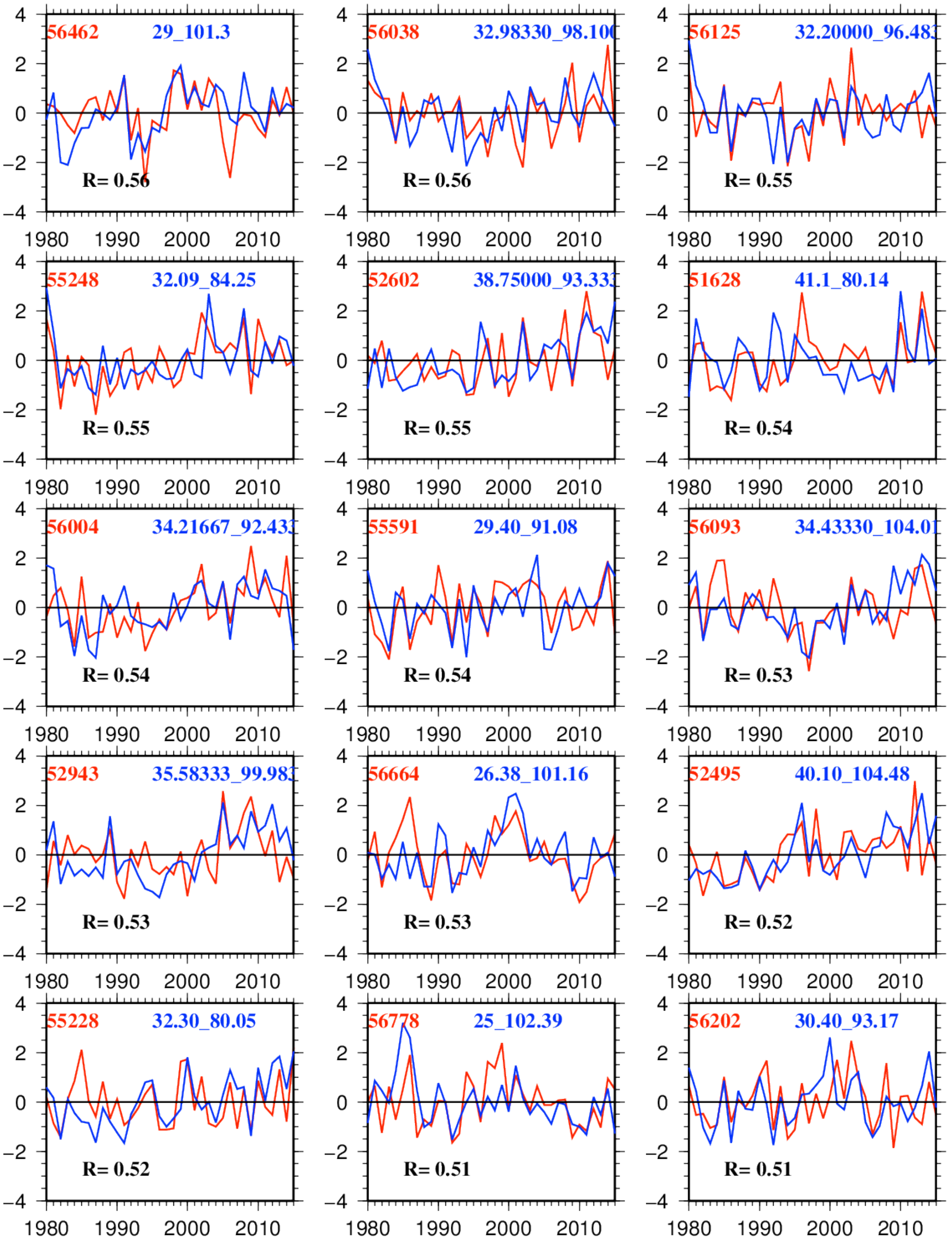

Figure S9. Continued. This figure was plotted using the Generic Mapping Tools (GMT) V4.5.0 (URL: https://www.soest.hawaii.edu/gmt/).

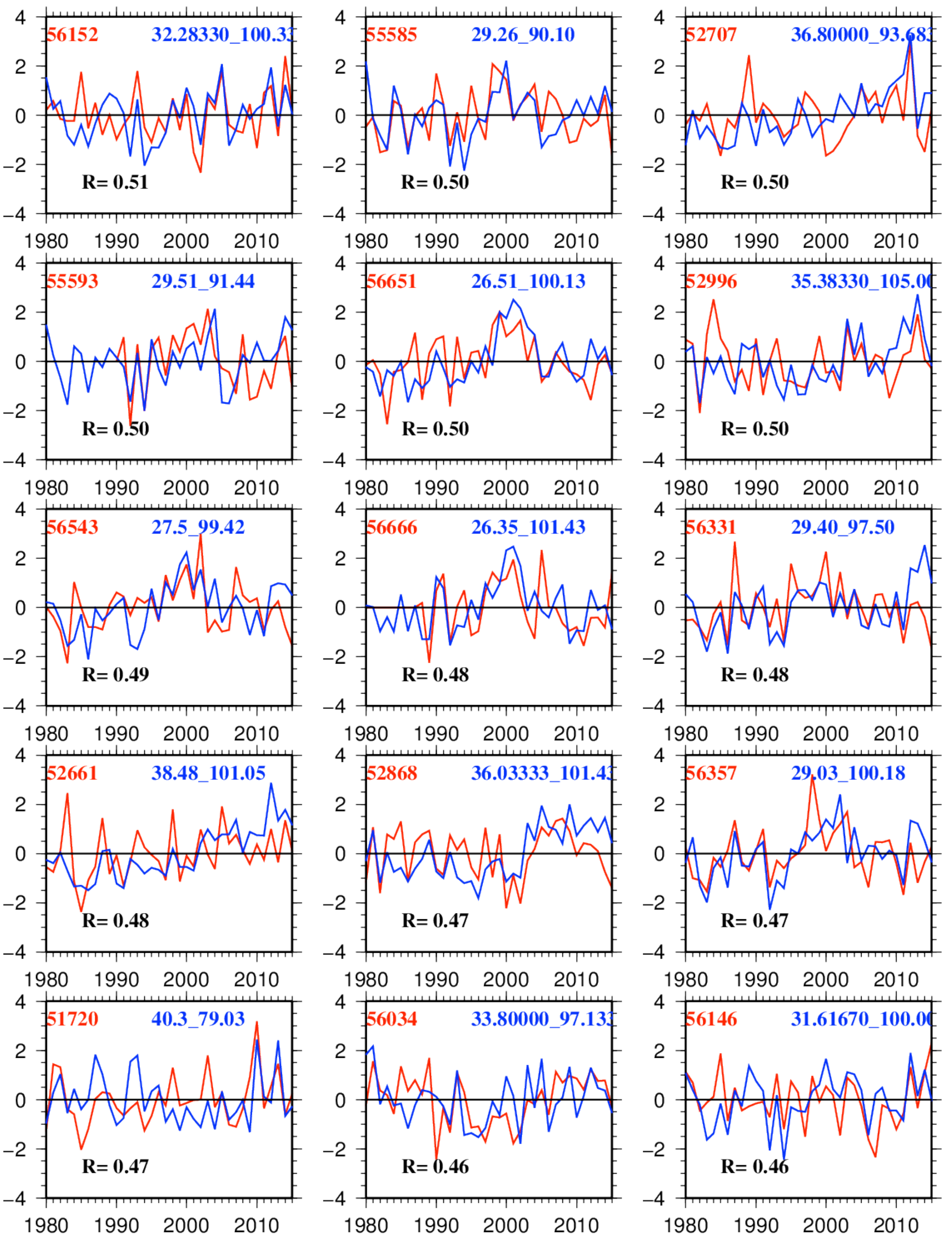

Figure S9. Continued. This figure was plotted using the Generic Mapping Tools (GMT) V4.5.0 (URL: https://www.soest.hawaii.edu/gmt/).

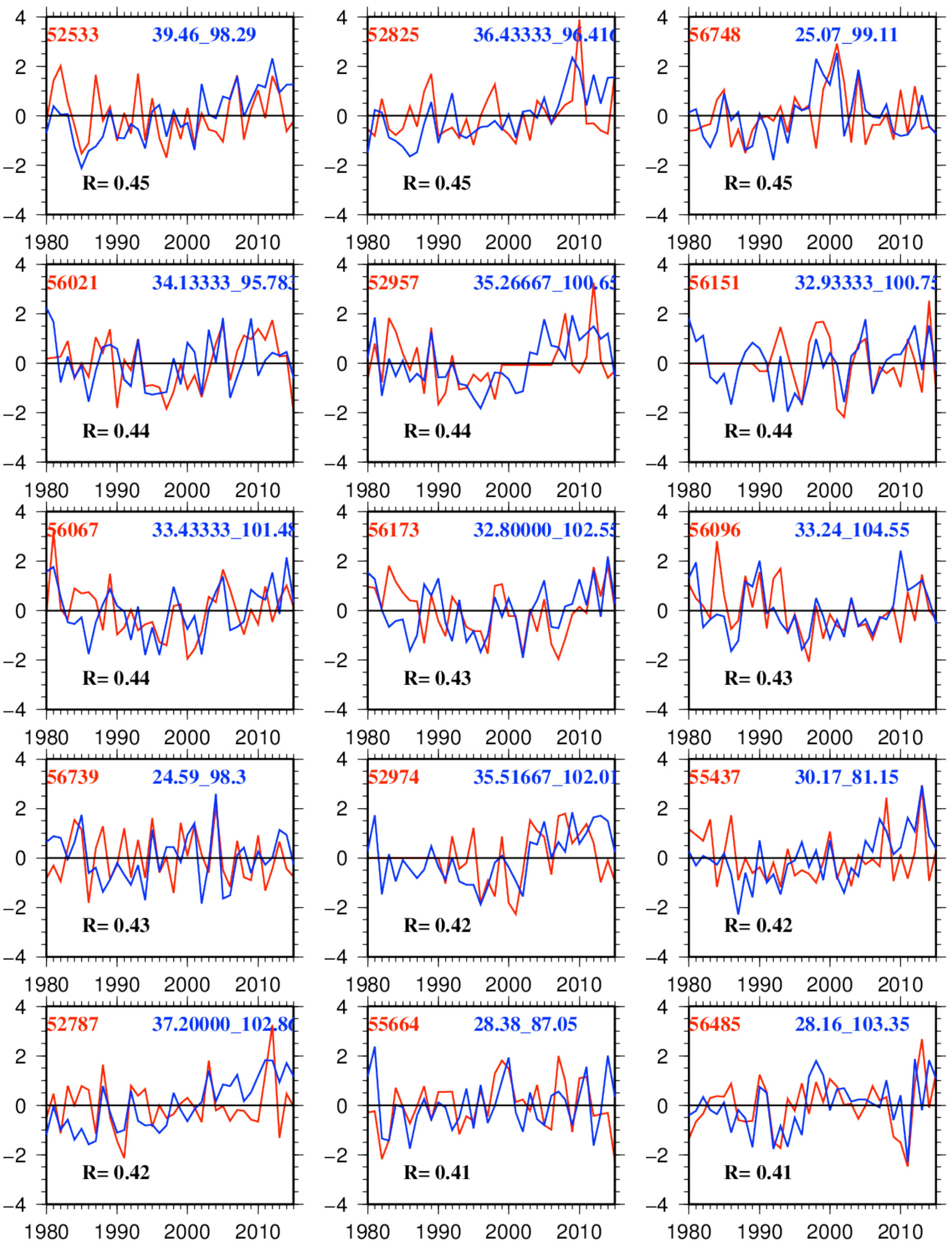

Figure S9. Continued. This figure was plotted using the Generic Mapping Tools (GMT) V4.5.0 (URL: https://www.soest.hawaii.edu/gmt/).

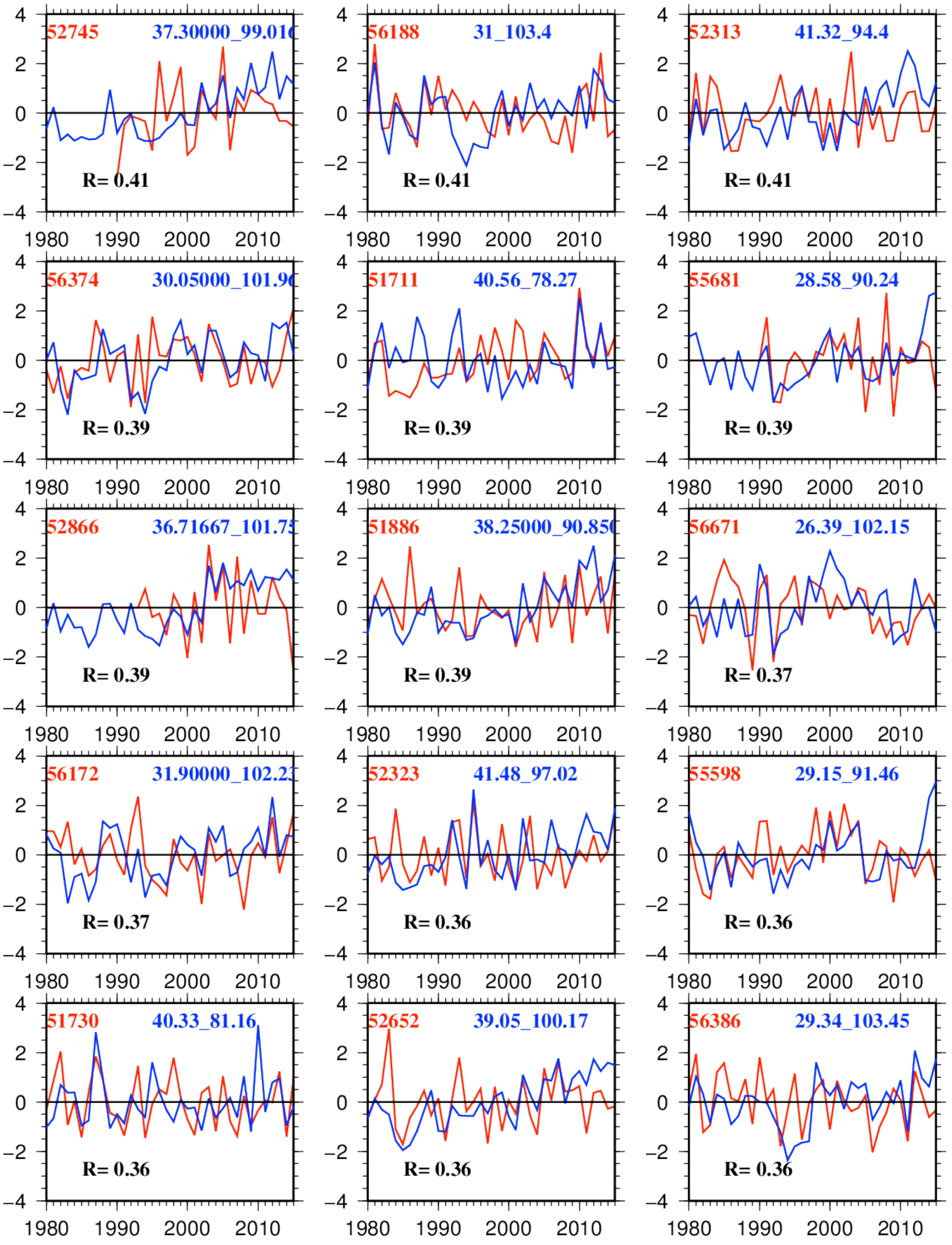

Figure S9. Continued. This figure was plotted using the Generic Mapping Tools (GMT) V4.5.0 (URL: https://www.soest.hawaii.edu/gmt/).

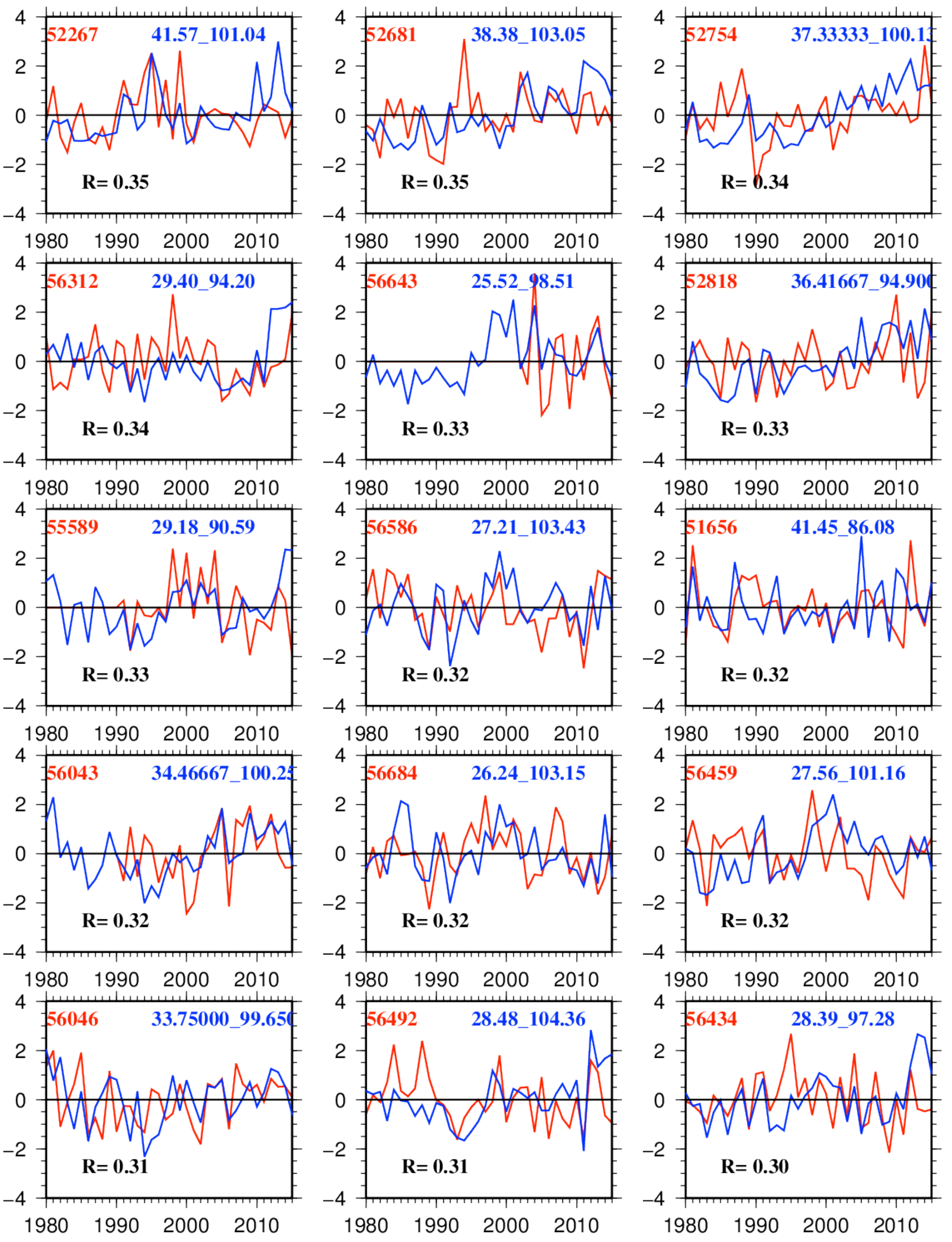

Figure S9. Continued. This figure was plotted using the Generic Mapping Tools (GMT) V4.5.0 (URL: https://www.soest.hawaii.edu/gmt/).

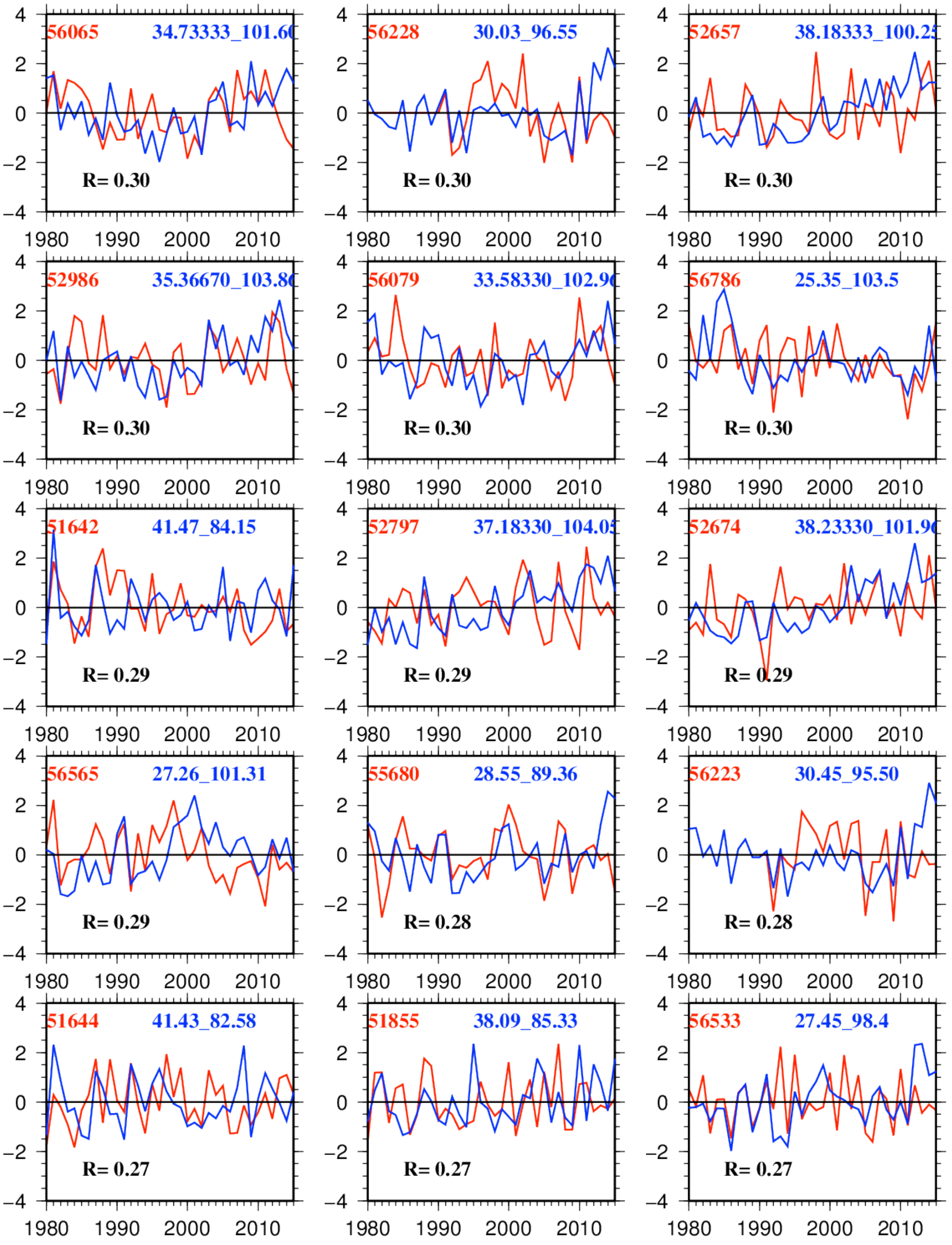

Figure S9. Continued. This figure was plotted using the Generic Mapping Tools (GMT) V4.5.0 (URL: https://www.soest.hawaii.edu/gmt/).

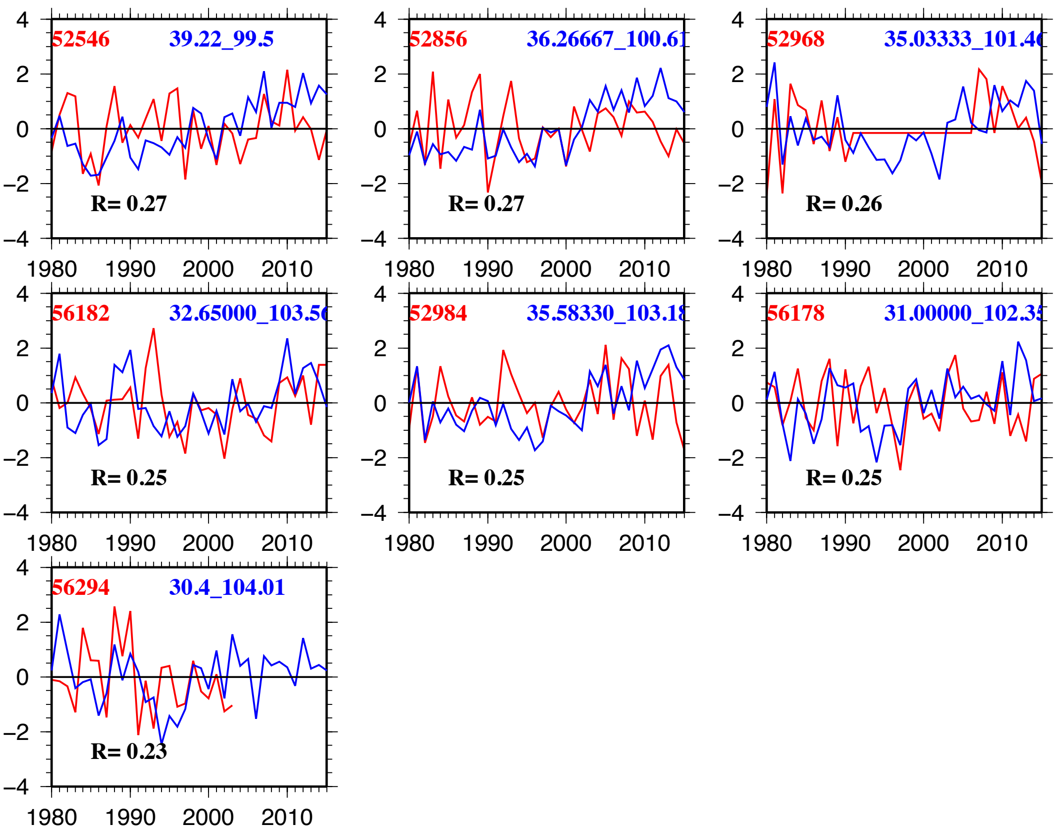

Figure S9. Continued. This figure was plotted using the Generic Mapping Tools (GMT) V4.5.0 (URL: https://www.soest.hawaii.edu/gmt/).
